# Fluid-layer-assisted mold temperature control for injection-molded bridge-type compliant mechanisms: Experimental thermal validation and simulation-based benchmarking against conventional cooling channels

**DOI:** 10.1371/journal.pone.0361383

**Published:** 2026-10-27

**Authors:** Pham Son Minh, Thanh Trung Do, Pham Ngoc Chuong, Hung-Son Dang

**Affiliations:** 1 Faculty of Mechanical Engineering, HCM City University of Technology and Engineering, Ho Chi Minh City, Vietnam; 2 Faculty of Energy Engineering and Transport, HCM City University of Technology and Engineering, Ho Chi Minh City, Vietnam; University of Sharjah, UNITED ARAB EMIRATES

## Abstract

The injection molding of bridge-type compliant mechanisms is sensitive to non-uniform mold temperature because the thin flexure hinges are prone to premature solidification and incomplete filling. This study evaluates a fluid-layer-assisted mold temperature-control architecture using experimental thermography, transient thermo-fluid simulation, a representative straight-channel thermal baseline, melt-filling analysis, and displacement testing of molded HDPE mechanisms. The fluid-layer thermo-fluid model was validated against infrared measurements, yielding a mean absolute error (MAE) of 5.01 °C and a root mean square error (RMSE) of 6.44 °C across six validation points. Under the numerical benchmark condition of an 80 °C inlet temperature, a mass flow rate of 59.5 g/s, and 40 s heating, the fluid-layer architecture was predicted to increase the average cavity-surface temperature by approximately 4.0 °C and reduce the maximum longitudinal temperature difference by 28.6% relative to the straight-channel model. Independent thermography of a representative steel straight-channel insert under the same inlet-temperature and flow-rate conditions showed a lower and more non-uniform surface-temperature field than the steel fluid-layer configuration, confirming the direction of the predicted thermal advantage. Melt-filling simulations subsequently predicted incomplete filling at the thin flexure region for the straight-channel configuration and complete filling for the fluid-layer configuration; this filling comparison remains simulation-based. For HDPE blends containing 0–25 wt% recycled material, the molded mechanisms retained an approximately linear input–output displacement response over the investigated range. At an input displacement of approximately 175 µm, the mean output displacement decreased from 857 µm for virgin HDPE to 745 µm at 25 wt% recycled HDPE. The results support the fluid-layer architecture as a conventionally machinable approach for improving mold-surface thermal control, while polymer morphology, fatigue durability, and experimental straight-channel filling remain subjects for future work.

## 1. Introduction

Injection molding is the most widely adopted manufacturing process for fabricating polymer components with complex geometries, tight dimensional tolerances, and high production efficiency. However, product quality is dictated not only by the cavity geometry but also significantly by the heat transfer process and the flow behavior of the polymer melt. The mold temperature field directly controls the melt viscosity, solidification kinetics, molecular orientation, and residual stresses. Non-uniform temperature distribution can cause premature solidification in thin-walled sections, leading to defects such as short-shots, weld lines, warpage, and dimensional inaccuracies [1–11]. Temperature control is particularly important for compliant mechanisms, including bridge-type mechanisms. These mechanisms transmit motion through the elastic deformation of highly slender flexure hinges [12–17]. Temperature differences during injection molding can contribute to asymmetric shrinkage, stiffness imbalance, and changes in the displacement amplification ratio [18–22]. Accordingly, mold temperature control can directly influence the dimensional and mechanical performance of the mechanism. To enhance thermal regulation, various mold heating and cooling strategies have been investigated. Conventional straight cooling channels are prevalent due to their structural simplicity, low cost, and ease of machining [23–27]. However, they cannot conform to the curved or thin profiles of the mold cavity, resulting in uneven heat dissipation and the formation of localized hot spots. Conversely, conformal cooling systems fabricated via 3D printing (Additive Manufacturing) allow channels to closely follow complex geometric surfaces [28–38]. Nevertheless, this technology entails high manufacturing costs, extended production times, and difficulties in integration with standard mold bases. Other methods, such as rapid heat cycle molding (RHCM/variothermal) [39–44] or gas/induction heating [45–49], can improve surface quality but typically consume substantial energy and require complex operational cycles. Table 1 summarizes the advantages and limitations of current state-of-the-art mold temperature control technologies, thereby identifying the technological gap that needs to be addressed.

**Table 1:** State-of-the-art gap in mold temperature control technologies.

Technology	Thermal characteristic	Principal limitation	Cycle-time implication	Mold durability/ maintenance	Manufacturing cost
Straight cooling channel	Simple and robust; conventional machining [23–27].	Limited conformity to complex cavities; local temperature gradients may occur.	Conventional cycle; cooling time depends strongly on channel-to-cavity distance.	Generally durable and comparatively easy to inspect and maintain.	Low.
Conformal cooling	High potential temperature uniformity when channels follow the cavity [28–38].	Often requires metal additive manufacturing and post-processing.	Can shorten cooling time when appropriately designed; application dependent.	Internal channels may be more difficult to inspect, clean, or repair.	High.
Variothermal/ RHCM	Rapid cyclic heating and cooling can improve surface replication [39–44].	Higher system and control complexity; additional energy input.	Adds controlled heating/cooling stages; total cycle effect depends on implementation.	Repeated thermal cycling can increase thermal loading of the tooling.	High.
Gas/ induction heating	Very rapid surface heating [45–49].	Heating can be localized and requires specialized auxiliary equipment.	Heating stage can be short, but auxiliary switching/control is required.	Maintenance depends on coils, gas delivery, insulation, and auxiliary hardware.	Medium to high.
Proposed fluid layer	Short local heat-transfer path and large fluid-solid interface; conventionally machinable.	Broader multi-cycle and production-scale validation is still required.	Not quantified in the present study.	Long-term mold durability was not quantified; modular plates can be disassembled for service.	Moderate; conventional CNC machining and external temperature-control hardware.

Consequently, the unresolved question is not simply whether a thinner fluid layer can enhance heat transfer, but whether a fluid-layer-assisted mold architecture can provide measurable thermal and molding advantages over a conventional straight-channel design under equivalent operating conditions. To address this gap, this study proposes a fluid-layer-assisted mold temperature control architecture in which a contoured interface and an adjacent fluid layer are used to shorten and homogenize the heat-transfer path while preserving manufacturability using conventional machining methods. The proposed architecture is evaluated against a conventional straight-channel configuration under identical operating conditions, with particular emphasis on cavity-surface temperature during transient heating, spatial temperature uniformity, mold-filling capability, and the displacement response of bridge-type compliant mechanisms. The investigation examines whether the fluid-layer design can achieve a higher cavity-surface temperature and a smaller maximum longitudinal temperature difference, thereby improving thermal uniformity across the cavity surface. The resulting thermal conditions are further assessed in terms of their ability to promote complete filling of the thin flexure hinges and to produce molded bridge-type compliant mechanisms with a consistent input–output displacement response over the investigated range. In addition, the functional performance of completely filled mechanisms is evaluated using HDPE containing different fractions of recycled material to characterize the short-term input–output displacement response across the investigated material compositions.

## 2. Materials and methods

### 2.1. Mold heating and cooling system

In injection molding, rapid and stable control of the cavity surface temperature is critical to product quality and process repeatability. To meet this requirement, a controlled mold heating system was developed to provide and maintain a predefined thermal field before and during the injection stage. The primary objective of the system was to establish reproducible thermal conditions while isolating the influence of the heat source from the dynamic behavior of the polymer melt. In this design, the heating system delivers thermal energy to the mold in a controlled manner, minimizes temperature fluctuations, and ensures that the cavity surface reaches the target thermal state prior to melt filling. This approach enables independent investigation of mold thermal responses under well-defined heating conditions. The system was designed based on a closed-loop thermal control architecture, in which the heat transfer medium continuously circulates between the heating unit and an integrated channel network within the mold. The closed-loop design reduces energy losses, stabilizes flow pressure, and enables precise regulation of the inlet temperature throughout the operating cycle. The overall structure consists of a mold heating unit, a circulation pump, control valves for flow routing, connecting pipelines, a flow meter, and temperature sensors. These components form a unified circulation loop, allowing the heat transfer medium to pass through the mold and return to the heating source continuously. During the heating mode, water was selected as the heat transfer medium due to its high specific heat capacity and operational stability. The water was heated to a preset temperature in the mold heating unit and then pumped through the system. Before entering the mold, the flow rate was monitored using a flow meter to ensure stable operating conditions. As the medium flows through the cooling layer, heat exchange occurs via forced convection on the fluid side and conduction within the solid mold domain, enabling the cavity surface to rapidly reach the desired temperature. After completing the heat exchange process, the medium exits through the outlet and returns to the heating unit, maintaining continuous thermal supply. In addition to temperature control, the system supports real-time thermal monitoring to ensure stability and repeatability of experimental conditions. The inlet and outlet temperatures of the circulating medium were measured using thermocouples, while the cavity surface temperature distribution was recorded using an infrared thermal imaging camera. The combination of contact and non-contact measurements allows simultaneous monitoring of both fluid and mold thermal behavior within the same time frame, providing a reliable data foundation for subsequent heat transfer analyses. The overall architecture of the heating and cooling system is illustrated in Fig 1.

**Fig 1:**
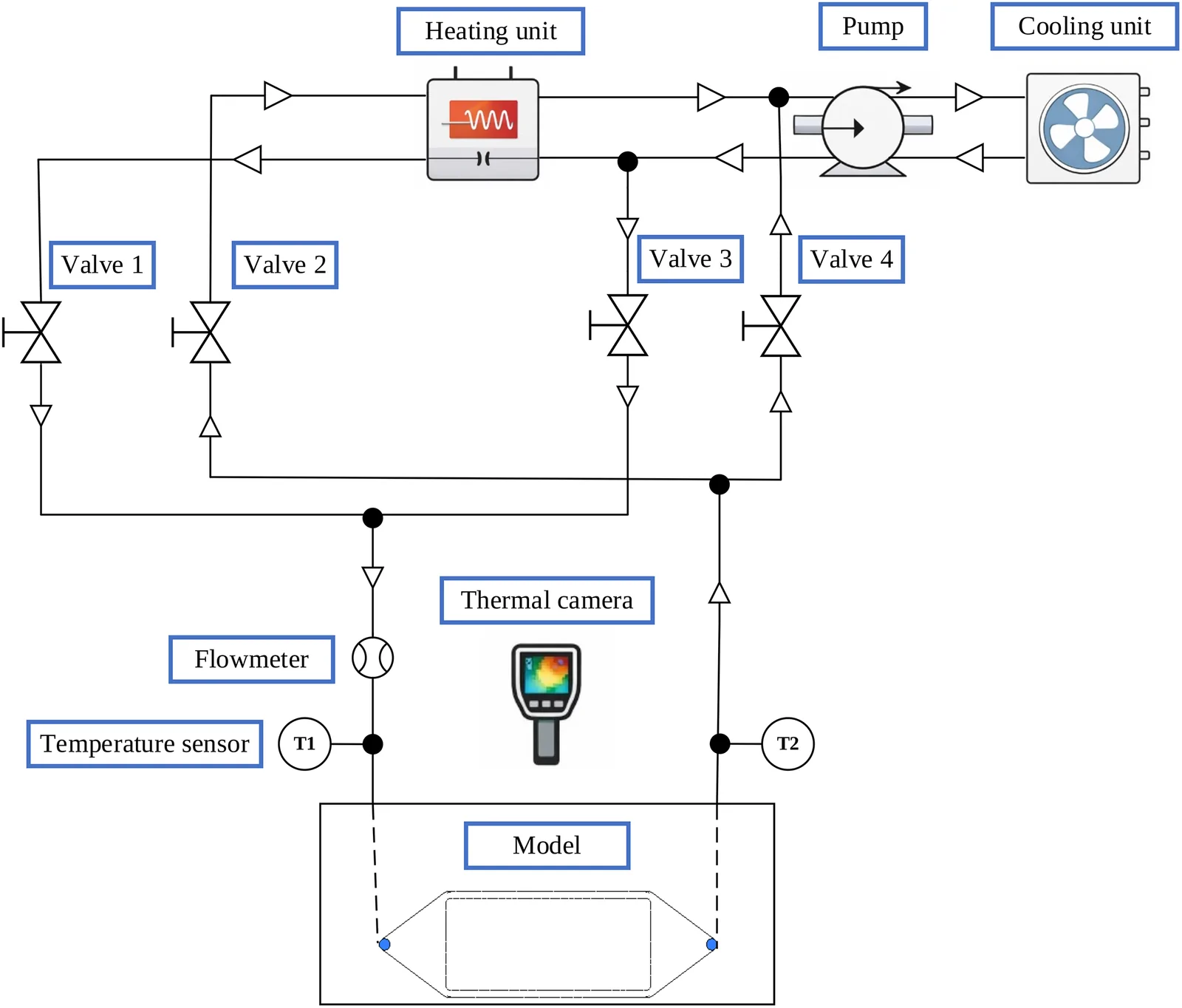
Schematic of the experimental system.

### 2.2. Operating principle of the integrated mold heating–cooling system

The operating principle of the integrated heating–cooling system was developed to utilize a single channel network within the cooling layer throughout all stages of the injection molding cycle, including mold heating, temperature holding during filling, and subsequent cooling. This approach ensures thermal continuity and reduces structural complexity compared with systems employing separate channels for heating and cooling. Prior to injection, the mold is brought to the target thermal condition under the heating mode. In this stage, the heat transfer medium, preheated in the mold heating unit, is circulated through the cooling layer at a controlled flow rate. As the fluid flows through the channels, heat is transferred to the mold structure via forced convection at the channel walls and conduction within the insert and mold body. Owing to the short heat transfer distance provided by the cooling layer, the cavity heating surface rapidly reaches the preset temperature, thereby establishing stable thermal conditions before melt filling. Once the target temperature is achieved, the system enters the temperature-holding phase. During this period, both the medium temperature and flow rate are maintained nearly constant to minimize thermal fluctuations prior to injection. When the polymer melt is injected into the cavity, the heat transfer medium continues to circulate within the cooling layer. The contact between the high-temperature melt and the cavity surface generates a significant interfacial heat flux. Continuous fluid circulation beneath the cavity surface enables efficient heat absorption and redistribution, preventing localized overheating and maintaining thermal uniformity during this critical stage of the molding cycle. After filling and initial solidification, the system switches to the cooling mode by redirecting the flow from the heating loop to the cooling loop through a valve-controlled mechanism. In this mode, chilled water supplied by a water chiller flows through the same channel network within the cooling layer, replacing the previously heated medium. Heat is extracted from both the mold structure and the molded part via forced convection and conduction. The use of a shared cooling layer for both heating and cooling ensures consistent heat transfer pathways across all stages, enabling comparable and repeatable thermal responses between cycles while reducing mechanical complexity and channel layout constraints.

A key feature of the operating principle is the fluid-switching mechanism controlled by a valve system. During heating and temperature holding, the cooling loop is completely isolated to eliminate interference from chilled water. Conversely, during cooling, the heating loop is disconnected to maximize heat extraction efficiency. After water cooling is completed, compressed air is introduced into the cooling layer channels to remove residual fluid, preventing water accumulation and minimizing corrosion risk. The complete sequence—heating, temperature holding with injection, cooling, and air purging—is implemented through this flow-switching strategy, as illustrated in Fig 2: heating, injection with temperature holding, cooling, and air purging. This operating principle provides the foundation for flexible and reproducible thermal control in the integrated cooling-layer mold system.

**Fig 2:**
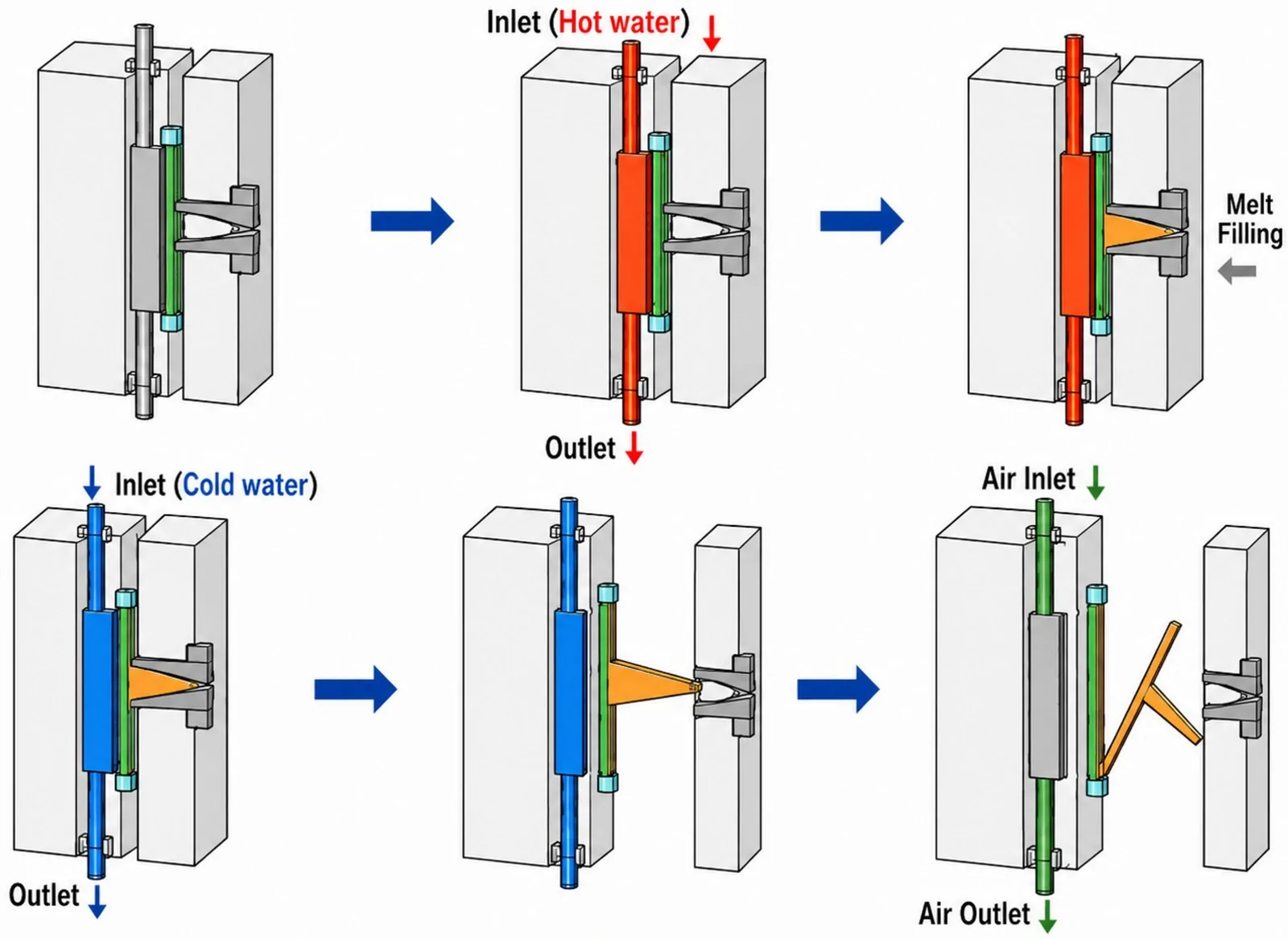
Schematic illustration of fluid switching during heating, injection with temperature holding, cooling, mold opening, and air purging.

### 2.3. Design setup of the experimental mold system

The experimental mold system was developed based on an insert–cooling plate architecture featuring a concave interface profile to enhance heat transfer control from the circulating medium to the cavity surface. Instead of using a conventional solid mold structure, a modular design was adopted, in which the insert plate carrying the cavity geometry was directly assembled onto a cooling plate through bolts and fluid connectors. This approach enables integration of a thermal distribution layer immediately beneath the forming surface while maintaining compatibility with standard injection molding machines. The primary design objective was to shorten the heat transfer path and establish a reproducible thermal field on the cavity surface without significantly altering melt filling conditions. As illustrated in Fig 3, the structure consists of two main components: the insert plate and the cooling plate. The insert plate, positioned on the top, contains the cavity surface in direct contact with the polymer melt. The cooling plate, located beneath the insert, accommodates the inlet and outlet connectors for the heat transfer medium. The two plates are fastened using a uniformly distributed bolt system along the perimeter, ensuring stable mechanical and thermal contact at the interface. The bolted assembly not only provides sufficient structural rigidity under injection pressure but also reduces thermal contact resistance between the insert and cooling plates, thereby enhancing heat conduction from the cooling layer to the cavity surface. Fluid connectors are arranged on the underside of the cooling plate to interface with the external heating–cooling system, enabling direct delivery of the medium to the primary heat transfer region. A key design feature is the concave profile formed at the contact interface between the insert and cooling plates. This profile follows the cavity geometry, maintaining a relatively uniform distance between the cavity surface and the underlying heat transfer domain. As a result, the thermal path from the cooling plate to the insert surface is shortened and homogenized compared with conventional flat designs, reducing lateral thermal gradients across the cavity surface. The water thickness parameter t was varied as 3, 5, and 8 mm in the parametric configurations to examine its influence on the thermal response while maintaining adequate structural stiffness under injection loading. The cooling plate functions as both a heat distribution and heat extraction layer. The concave geometry shapes the thermal field in accordance with the cavity contour, while the symmetric layout and circumferential bolt arrangement minimize assembly deformation under combined thermal and mechanical loads. The overall dimensions of the insert–cooling plate assembly are approximately 350 mm × 150 mm, providing sufficient area for stable thermal distribution while remaining compatible with the experimental mold frame. The cavity is centrally positioned to reduce edge effects and promote symmetrical thermal development along both length and width directions. Overall, the concave insert–cooling plate design establishes a short and controllable heat transfer architecture for injection molding applications. The combination of bolted assembly, stable thermal contact, and geometry-conforming interface ensures mechanical robustness under injection pressure while enabling effective thermal regulation. This design, shown in Fig 3, forms the basis for the subsequent numerical simulations and experimental investigations of mold thermal behavior.

**Fig 3:**
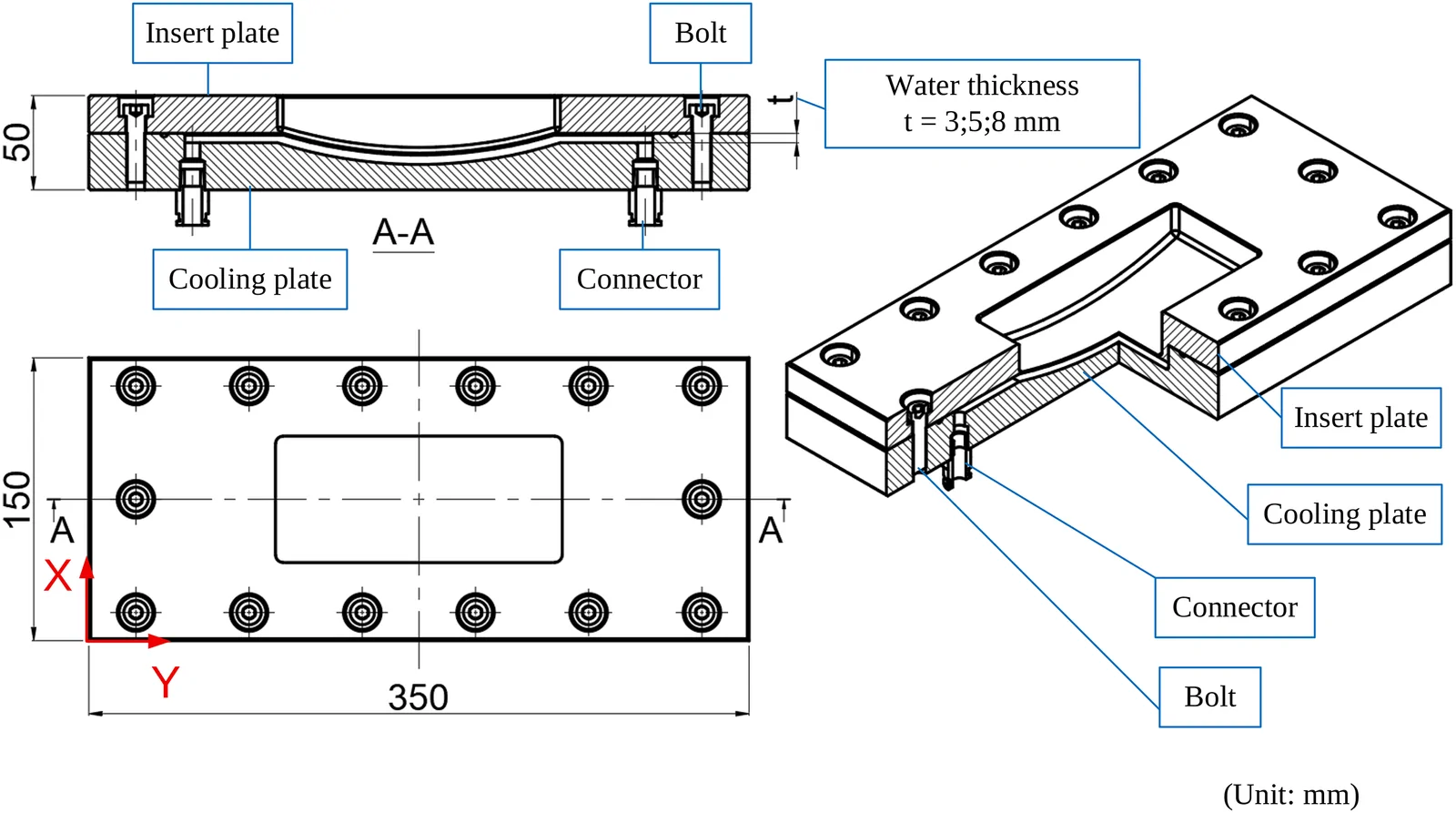
Structural model of the insert and cooling plate system.

### 2.4. Numerical simulation model

A numerical model was developed to analyze the transient thermal response of the contoured insert plate–cooling plate system and to elucidate the heat transfer mechanism from the circulating medium within the cooling layer to the cavity surface prior to and during injection molding. The primary focus of the model is the internal mold temperature field, allowing the isolation of the cooling layer architecture and mold insert design’s influence from the kinematic behavior of the polymer melt. The component layout and computational domain design of the system are detailed in Fig 4. The simulation serves as an analytical tool to explain temperature distribution characteristics and provides a quantitative basis for comparison with experimental measurements. The simulation geometry was constructed based on the physical insert plate–cooling plate structure. As shown in the 3D structural schematic in Fig 4, the computational domain includes solid regions (insert plate and cooling plate) and a fluid region representing the heat transfer medium within the cooling layer. To reduce computational costs while preserving essential thermal characteristics, non-critical geometric features—such as threads, bolts, and minor chamfers—were omitted. However, the contoured contact surface and the distance between the fluid domain and the cavity surface were retained to ensure the accurate representation of thermal conduction resistance. Heat transfer in the solid domains is governed by the transient heat conduction equation, with material properties—including thermal conductivity, density, and specific heat capacity—assigned according to the mold material. In the fluid domain, heat exchange is described by conjugate heat transfer (convection-conduction) formulations, assuming fully developed flow within the channels. This approach captures the thermal interaction between the circulating medium and the mold structure without necessitating the resolution of the entire supply system’s flow field. Boundary conditions were defined according to the operating modes of the integrated heating-cooling system. At the solid-liquid interface, coupled thermal boundary conditions were applied to ensure consistent temperature and heat flux continuity. The inlet temperature of the fluid domain was defined based on the preset values of the mold temperature controller or chiller, while the mass flow rate was imposed as the inlet-flow condition and therefore governed the convective transport in the resolved fluid domain. The external surfaces of the mold exposed to ambient air were assigned natural convection boundary conditions to account for environmental heat loss. During the pre- injection heating phase, the cavity surface was also simulated with air convection boundary conditions to evaluate the mold’s thermal behavior independently of polymer effects. The computational domain was discretized using a heterogeneous mesh, with elements refined in regions exhibiting high temperature gradients, particularly near the insert plate– cooling plate interface and the cavity surface. Transient simulations were conducted to record the temporal evolution of temperature during the heating and holding phases. A formal three-level mesh and time-step independence study was not available in the archived numerical dataset; therefore, no quantitative discretization-independence claim is made here. Key outputs include the cavity surface temperature distribution, through-thickness thermal gradients within the insert plate, and the heat flux transferred from the fluid domain to the mold. These quantities were extracted over time for comparison with data from the infrared camera and thermocouples acquired in the experimental setup, as described in Fig 4. The numerical model complements the experiments by providing information on internal temperature gradients and heat-transfer trends that cannot be obtained directly from surface measurements. The combined simulation-experimental approach allows for a consistent evaluation between theoretical predictions and the actual thermal behavior of the contoured cooling layer mold system. This research framework establishes the foundation for subsequent analyses of the relationship between heat transfer structure and thermal control performance in injection molding applications. To systematically validate the transient conjugate convection-conduction heat transfer simulation model against infrared thermography experiments, two distinct case studies regarding geometry and operating parameters were established. Design 1 represents the baseline low-intensity thermal condition, featuring an insert thickness of 1 mm, a fluid layer thickness of 3 mm, an inlet temperature of 60 °C, and a cooling water flow rate of 51 g/s. Design 2 represents a higher-temperature validation condition, with an insert thickness of 0.5 mm, a fluid-layer thickness of 10 mm, an inlet temperature of 80 °C, and a water flow rate of 59.5 g/s. These two distinct conditions were used to evaluate the numerical model over different geometric and operating conditions before the subsequent parametric and benchmark analyses.

**Fig 4:**
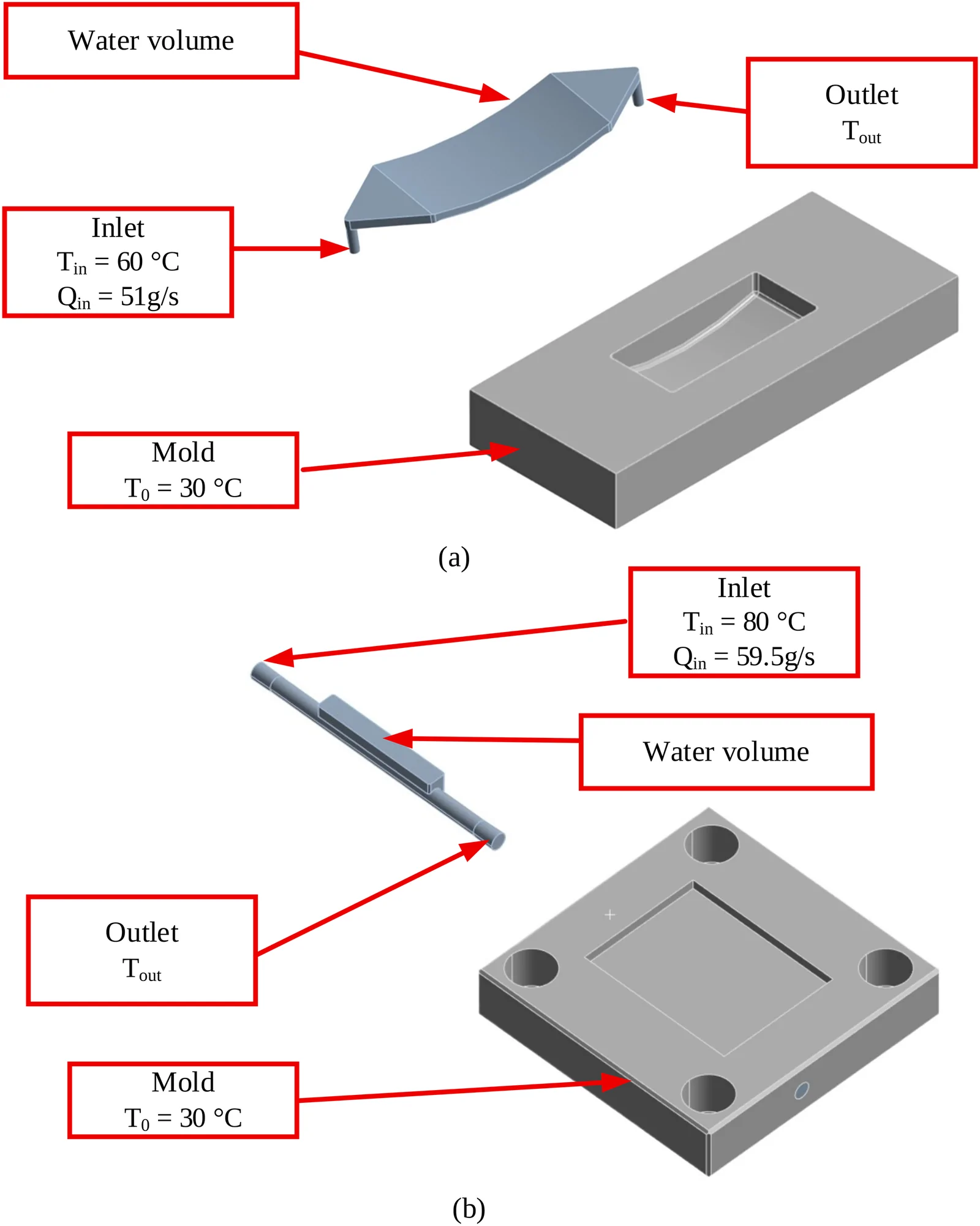
3D structural components and boundary designs of the fluid-layer-assisted mold: (a) Design 1 (Simple model); (b) Design 2 (Application model).

#### 2.4.1. Governing equations and boundary conditions.

The thermo-fluid model was formulated using conservation of mass, momentum, and energy in the water domain coupled to transient heat conduction in the steel mold domains. In compact form, the governing equations are:


$$\begin{array}{c} \partial \rho /\partial t\;+\;\nabla \cdot (\rho u=\;\text{0} \end{array}$$

$$\begin{array}{c} \rho [\partial u/\partial t\;+\;(u\cdot \nabla )u]\;=\;-\nabla p\;+\;\nabla \cdot \{\mu [\nabla u\;+\;(\nabla u{)}^{T}\} \end{array}$$

$$\begin{array}{c} \rho c_{p}[\partial T/\partial t\;+\;u\cdot \nabla T]\;=\;\nabla \cdot (k\_f\nabla T) \end{array}$$

$$\begin{array}{c} \rho \_s\;c_{p},s\;\partial T\_s/\partial t\;=\;\nabla \cdot (k\_s\nabla T\_s) \end{array}$$

At the resolved fluid–solid interface, temperature and normal heat flux were continuous. The measured mass flow rate and prescribed inlet-water temperature defined the inlet condition, while the outlet provided the downstream flow boundary. External mold surfaces exposed to laboratory air were represented by ambient convective heat loss. The mass flow rate therefore controls the convective transport through the resolved fluid solution rather than serving as an independently prescribed heat-transfer coefficient at the fluid–solid interface. The archived source model represents the insert/cooling-plate thermal interface as thermally continuous; a separate experimentally measured contact-resistance value was not available for this revision.

The original archived files available for this revision do not provide a complete solver-specific record of turbulence-closure settings or a formal three-level mesh/time-step independence study. Accordingly, the revised manuscript reports the documented governing formulation and boundary conditions, removes the previous unsupported statement that formal grid independence had been quantitatively verified, and treats quantitative discretization sensitivity as a limitation requiring additional numerical work.

### 2.5. Experimental measurement setup and data acquisition

#### 2.5.1. Temperature measurement setup.

In injection molding, the thermal state of the mold cavity prior to filling directly influences surface replication quality, residual stress development, and the dimensional stability of the molded part. In this study, temperature measurement is not treated merely as a collection of discrete data points but as a systematic characterization of the overall thermal response of the heated cavity surface during controlled heating and temperature maintenance phases. The primary objective is to isolate the impact of the integrated heating-cooling system from the complex heat exchange mechanisms that occur when the polymer melt contacts the mold. This approach enables a clear evaluation of the mold’s thermal control capability, particularly for the contoured cooling layer design. To ensure consistency, the measurement strategy is aligned with the numerical model, encompassing the spatial domain design and the selection of representative surface locations. To accurately represent the physical heat transfer path, the thermal domain in both the simulation and experiments is structured to maintain a continuous, meaningful heat path from the circulating medium to the cavity surface. The mold insert plate region, which directly supports the heated cavity surface, is identified as the critical zone governing the mold’s thermal behavior. Therefore, spatial resolution in this region is prioritized to capture through-thickness temperature attenuation and near-surface thermal gradients with high fidelity. This design permits precise profiling of heat accumulation and distribution across the entire bridge-type cavity throughout the heating and holding phases, providing a reliable basis for correlating numerical predictions with experimental observations.

The computational domain design and meshing strategy applied to the measurement-oriented model are illustrated in Fig 5. Instead of purely geometry-based discretization, the material domains—including the insert plate, the cooling water layer, and the mold base—are explicitly separated to accurately assign their respective thermal roles. In the insert plate region, the mesh is refined along the curved profile of the cavity to capture the steep thermal gradients developing between the heated cavity surface and the underlying metal layer. This refinement minimizes computational error in extracting surface temperatures and ensures that the predicted temperature field can be directly compared with experimental measurements.

**Fig 5:**
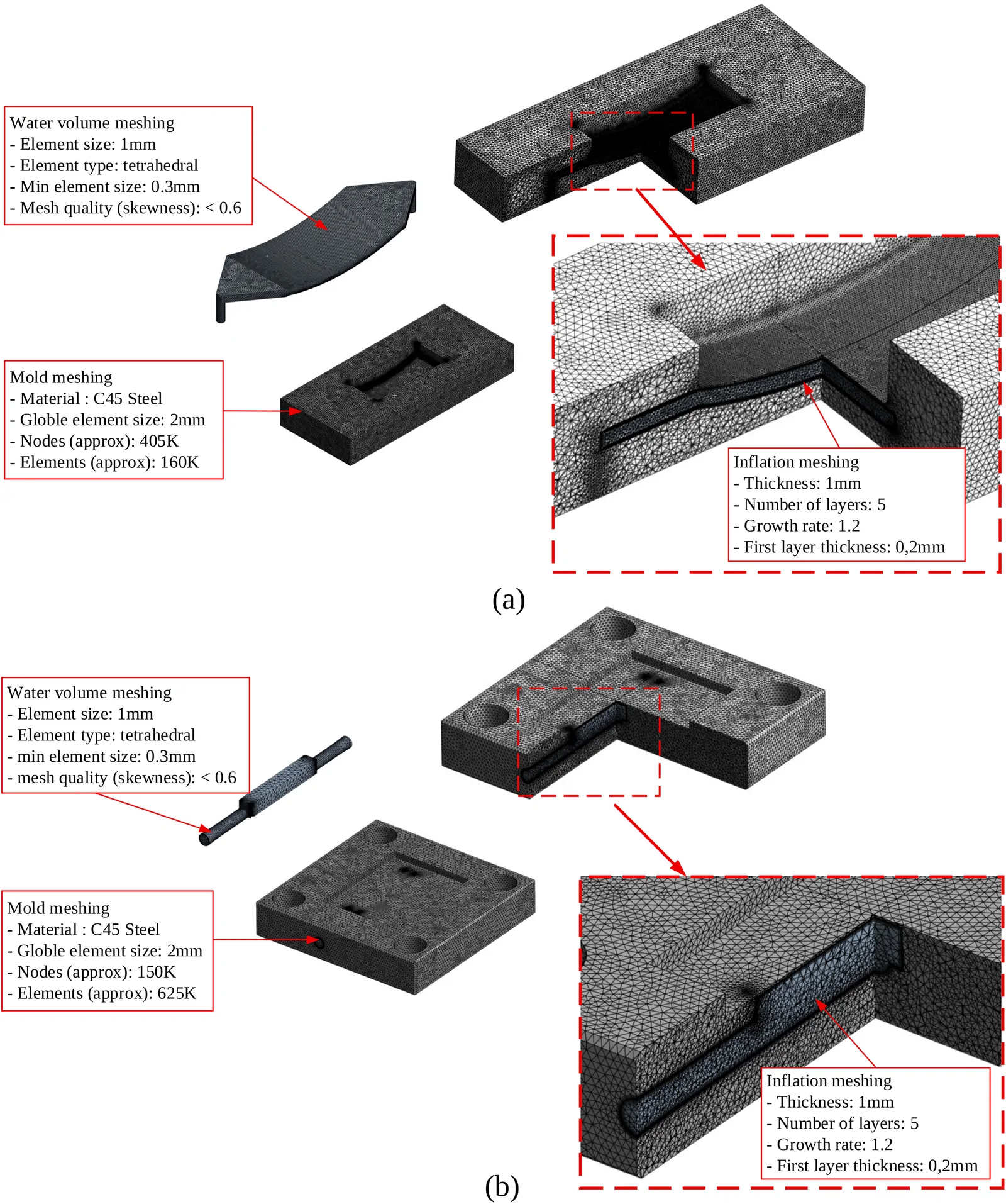
Meshing strategy and geometric cross-sections of the simulation model: (a) Design 1 and (b) Design 2.

Beneath the insert plate, the cooling water layer acts as an intermediate heat transfer zone between the heat source and the cavity surface. Rather than treating it simply as a fluid passageway, this region is simulated to represent the conjugate conduction-convection coupling between the circulating water and the channel walls. The mesh density in this region is selected to adequately resolve the thermal boundary layer forming along the channel surfaces, as this layer governs the heat exchange rate between the fluid and the mold structure. Conversely, the mold base primarily participates in bulk heat conduction and thus requires only moderate spatial resolution, sufficient to maintain numerical stability without incurring excessive computational costs.

At the interface between the insert plate and the cooling plate, thin inflation layers (prism layers) are inserted perpendicular to the interface to accurately capture through-thickness thermal gradients. These layered elements allow for an explicit representation of heat transfer from the heated cavity surface, through the insert plate, and into the cooling layer. Consequently, the temperature attenuation across the metal thickness is modeled as a physically continuous heat conduction process rather than a mere numerical interpolation, thereby enhancing the reliability and interpretability of the simulation results.

Parallel to the numerical model, the layout of measurement locations on the physical mold was designed based on the same fundamental physical principles, as illustrated in Fig 6. Cross-section A–A highlights the spatial relationship between the heated cavity surface, the insert plate, and the underlying cooling water layer. The curved profile of the bridge cavity not only defines the product geometry but also dictates the heat transfer distance from the molding surface to the cooling channels. Accordingly, the cooling water layer is positioned relatively close to the cavity surface to shorten the thermal path, while maintaining adequate mechanical strength for the insert plate under actual injection molding conditions.

**Fig 6:**
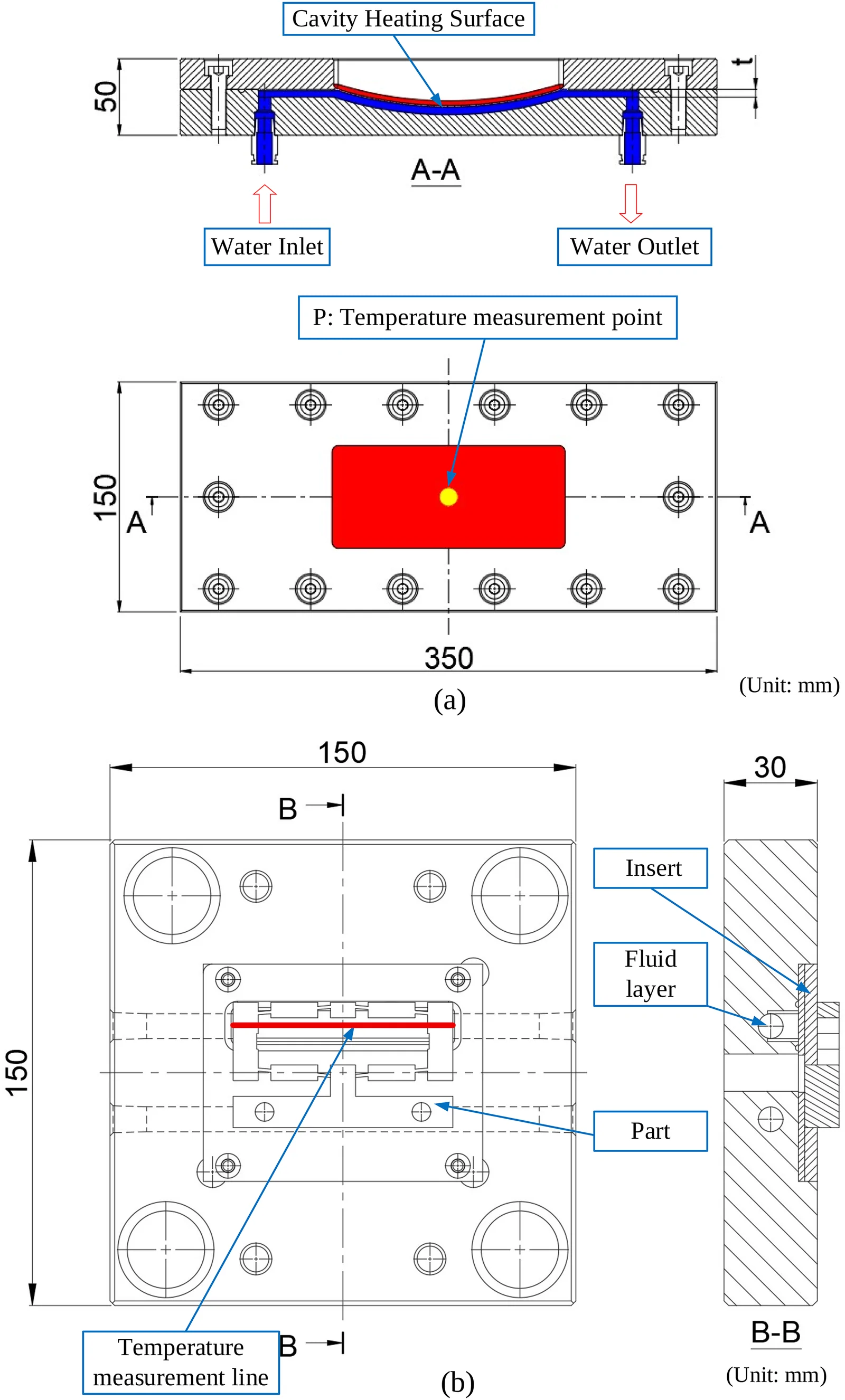
Schematic of the cooling water layer and the temperature measurement strategies: (a) Point-based measurement (Point *P*) for Design 1; (b) Line-based profile measurement for Design 2.

From a fluid dynamics perspective, the heat transfer medium enters via the inlet and exits at the outlet, establishing a flow path along the length of the cavity. This design ensures uniform sweeping of the heat transfer zone and minimizes the potential for localized inefficient heat exchange areas (dead zones). In the top-down view, the central region representing the heated cavity surface will directly contact the polymer during injection. To most thoroughly characterize the thermal response, two distinct measurement strategies were applied to each case study to optimize data acquisition. For Design 1, a representative location (Point P) was selected precisely at the center of the cavity to record the transient average thermal response. Meanwhile, for Design 2, a measurement line along the length of the cavity was established to capture the full spatial temperature profile (line profile). The geometric center of this measurement line coincides perfectly with Point P of Design 1, creating a consistent reference benchmark linking the simulated thermal field and the experimental data shown in Fig 6. Through this coordinated modeling and measurement approach, the acquired data goes beyond descriptive temperature reporting and supports the mechanistic interpretation of heat transfer within the contoured cooling layer mold system.

#### 2.5.2. Experimental setup.

In this study, the experimental instrumentation system was designed not only to supply and regulate the thermal conditions of the mold, but also to ensure stable and repeatable observation of the cavity’s thermal response. The instrumentation strategy extended beyond the installation of individual sensors and components; instead, it focused on establishing an integrated system in which the heating source, cooling source, measurement devices, and flow control elements operate in a coordinated manner to create a precisely controlled thermal environment for the injection mold. The overall design was developed to guarantee consistency between imposed thermal conditions and recorded temperature data, thereby enabling reliable correlation with numerical predictions. The complete experimental setup, including heating and cooling units, circulation system, sensing elements, and data acquisition components, is illustrated in Fig 7. The thermography used for thermal-model validation and architecture benchmarking was conducted during the controlled pre-injection mold-heating stage, without polymer in the cavity. This choice was intentional so that the thermal response of the mold and circulating-fluid system could be isolated from the transient heat input of the injected melt; the subsequent injection-molding trials were treated as a separate application stage.

**Fig 7:**
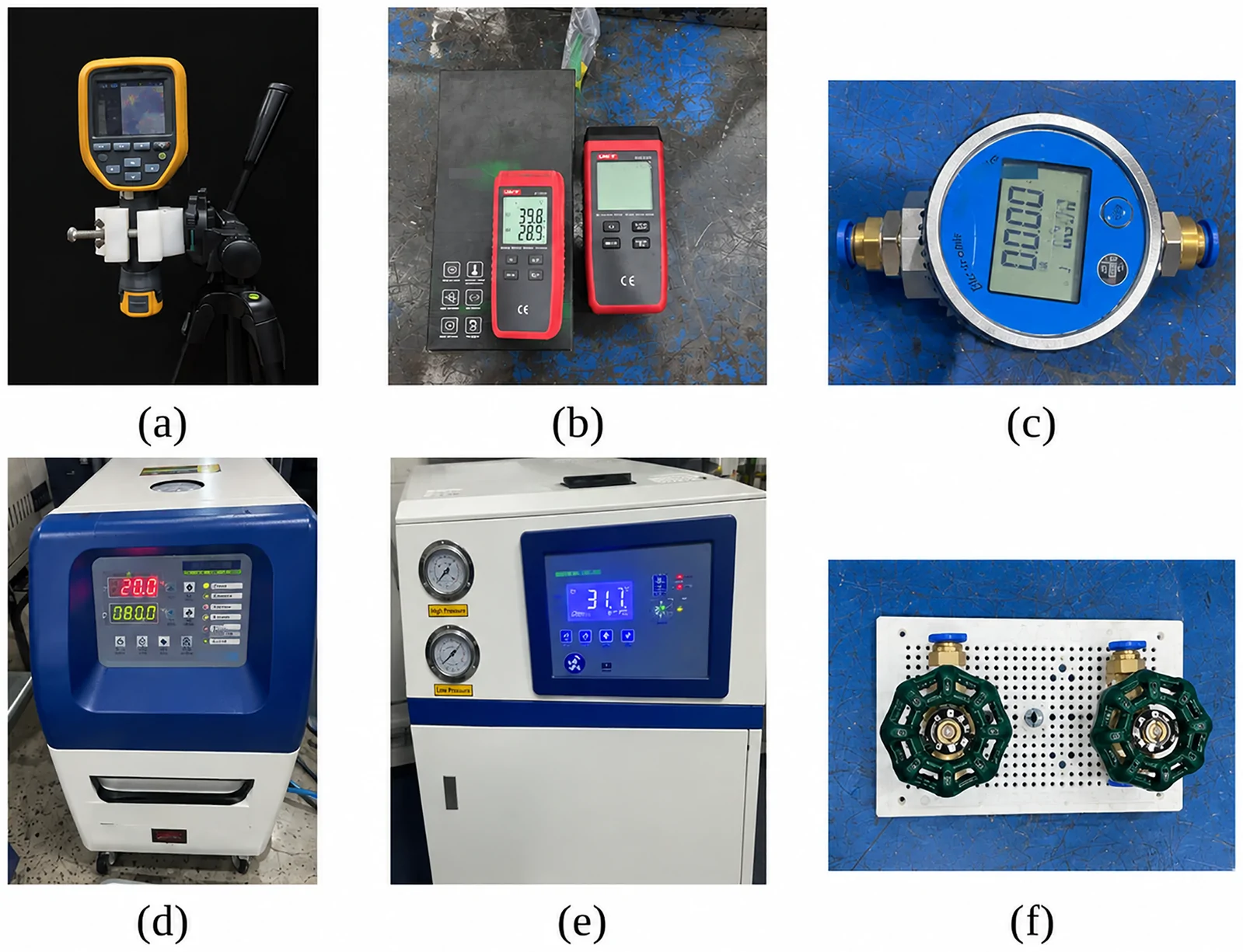
Experimental equipment used for temperature measurement: (a) infrared thermal camera, (b) dual-input thermometer, (c) water flow meter, (d) mold heating unit, (e) industrial water chiller, and (f) valves. (All photographs were taken by the authors in their laboratory).

In this system, the central component is the Mold Heating Unit (Haitian), which preheats the circulating medium before it enters the cooling water layer located beneath the insert. The unit enables precise temperature setting and maintenance, allowing the mold to reach and sustain the target thermal condition during the heating and temperature-holding phases. Instead of directly heating the mold using embedded electric heaters, the use of a circulating medium promotes more uniform thermal distribution within the cooling layer and reduces the risk of localized overheating within the insert. In parallel with the heating unit, an industrial water chiller was integrated to perform cooling functions when switching operational modes or restoring the mold to its initial condition. The chiller lowers the temperature of the circulating medium before it re-enters the mold, enabling flexible transition between heating and cooling phases. The combined use of the heating unit and chiller allows not only maintenance of a target temperature but also regulation of the medium’s temperature change rate, which is critical for ensuring thermal stability within the mold. To ensure appropriate flow conditions within the cooling layer, a water flow meter was installed along the supply line to monitor the flow rate. Flow rate data enable indirect control of the convective heat transfer intensity between the circulating water and the channel walls, thereby influencing the rate of heat transfer from the cooling layer to the insert. In addition, a system of valves was arranged along the piping branches to regulate flow direction and magnitude. This design permits flexible switching between the heating loop and cooling loop, as well as fine adjustment of the operating conditions during different experimental stages. For temperature measurement, both contact and non-contact methods were employed. A mini Type K/J dual-input thermometer was used to acquire signals from thermocouples installed at representative locations, including the fluid inlet, outlet, and mold body. Simultaneous monitoring of the medium temperature and mold metal temperature enables direct evaluation of the relationship between cooling layer operating conditions and the thermal response of the insert. This arrangement is particularly valuable during the heating phase, where the temperature difference between the circulating water and the mold metal governs the cavity heating rate. In addition to contact measurements, a Fluke TiS20 infrared thermal camera was used to observe real-time temperature distribution across the cavity heating surface. The non-contact measurement method allows full-field thermal visualization rather than discrete point readings, providing spatial information on temperature uniformity across the bridge-type cavity. The infrared camera is especially effective for detecting localized hot and cold regions arising from cavity geometry or flow characteristics within the cooling layer. The coordinated integration of these components, as shown in Fig 7, forms a comprehensive instrumentation system in which the circulating medium is thermally conditioned in a controlled manner, flow conditions are continuously monitored, and mold temperature fields are observed through both contact and non-contact sensing techniques. This design ensures stable and repeatable characterization of cavity thermal response while enabling direct correlation with the operating conditions of the integrated heating–cooling system, thereby providing a reliable basis for comparison with the numerical simulation results presented in subsequent sections. For the thermographic measurements used in the revised thermal benchmark, the steel measurement surfaces were coated with the same matte black finish. The infrared-camera emissivity setting was 0.93, the reflected-background temperature was 27.6 °C, and the transmission setting was 1.0. These settings were held constant for the compared steel inserts.

#### 2.5.3. Experimental straight-channel thermal benchmark.

A representative conventional straight-channel steel insert was evaluated experimentally to provide an independent thermal baseline for the proposed steel fluid-layer configuration. The straight-channel experiment used the same inlet-water temperature (80 °C), mass flow rate (59.5 g/s), and nominal heating duration (40 s) as the benchmark condition used in Section 3.2. The surfaces compared by infrared thermography were prepared with the same matte black coating, and the same Fluke TiS20 camera settings were retained. Radiometric IS2 data were recorded at 0, 5, 10, 20, 30, and 40 s. For the architecture comparison, the central cavity-surface temperature was extracted from a small central neighborhood, and a 70-mm longitudinal profile spanning the bridge-type molding region was extracted at the same registered surface location used for the fluid-layer line-profile analysis. The line-based non-uniformity was calculated as ΔTline = Tmax,line − Tmin,line. Because the archived straight-channel dataset contains one representative heating sequence rather than repeated independent runs, these measurements are reported as representative values and are not assigned standard-deviation error bars.

The purpose of this experiment was thermal benchmarking only. No new straight-channel injection-molding trials were performed for the present revision. Accordingly, the experimental straight-channel data are used to evaluate temperature response and spatial uniformity, whereas the short-shot/complete-filling comparison in Section 3.4 remains a numerical melt-filling result.

#### 2.5.4. Injection molding experimental setup.

To evaluate melt-filling behavior and the short-term displacement response of the molded parts under the investigated thermal-control conditions, a bridge-type compliant mechanism was selected as the experimental specimen. Fig 8 presents the detailed technical drawing and core geometric parameters of this mechanism.

**Fig 8:**
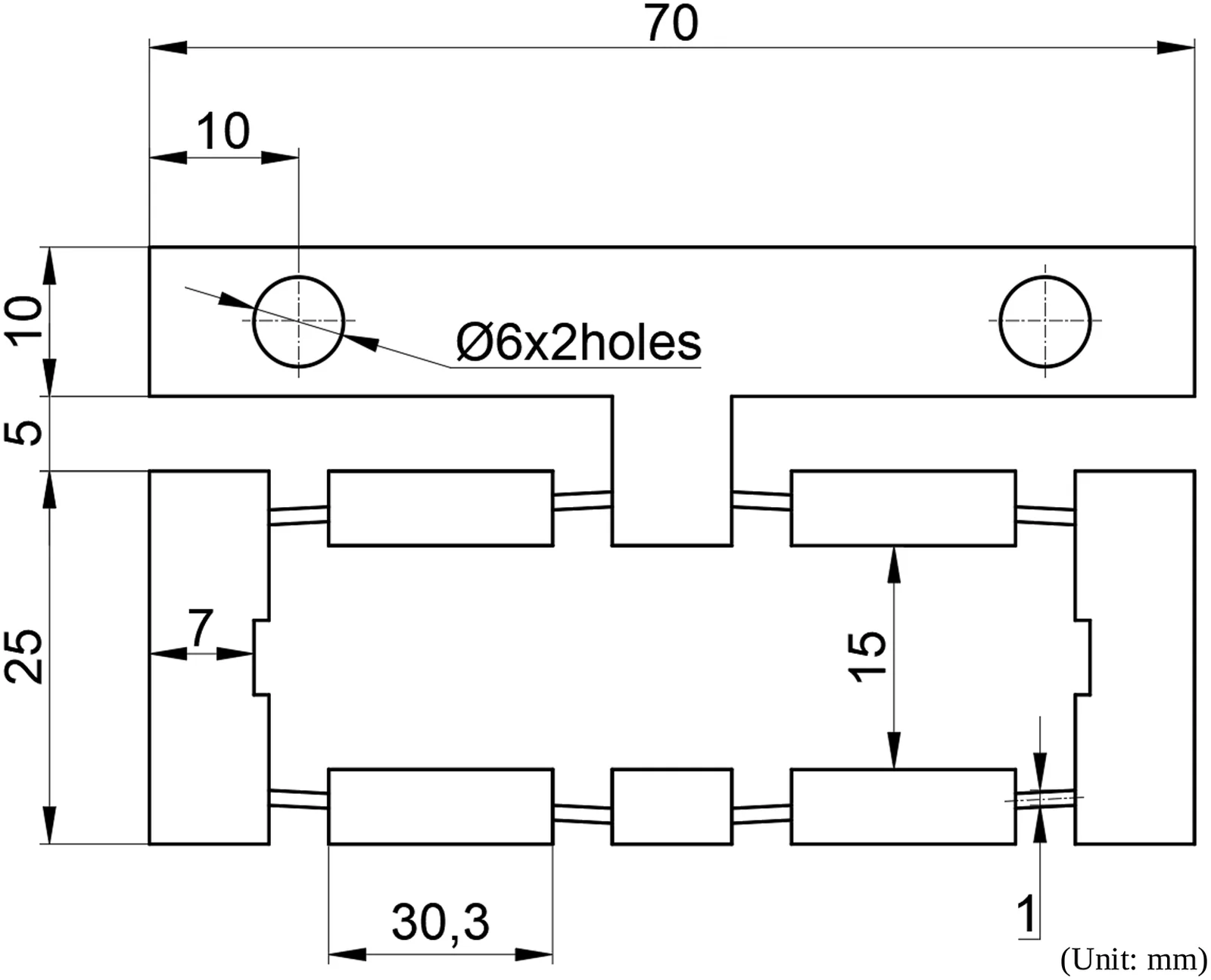
Geometric specifications and key dimensions of the bridge-type compliant mechanism selected for injection molding trials.

The primary rheological limitation and most significant manufacturing challenge of this design lie in the flexure hinges, where the wall thickness narrows to just 1 mm. During the filling phase of the injection molding cycle, this region acts as a hydrodynamic bottleneck, inducing substantial pressure losses and rendering the polymer melt flow highly sensitive to the mold wall temperature field. The experimental injection molding trials were conducted using a modular mold assembly structured around the insert–cooling plate assembly. Fig 9 illustrates the actual fabricated components of the insert and cooling plates after precision machining for both Design 1 and Design 2. The filling system uses a central sprue/gate located on the mechanism midline, providing a symmetric feed into the bridge-type cavity; this location corresponds to the central feed position used in the melt-filling analysis.

**Fig 9:**
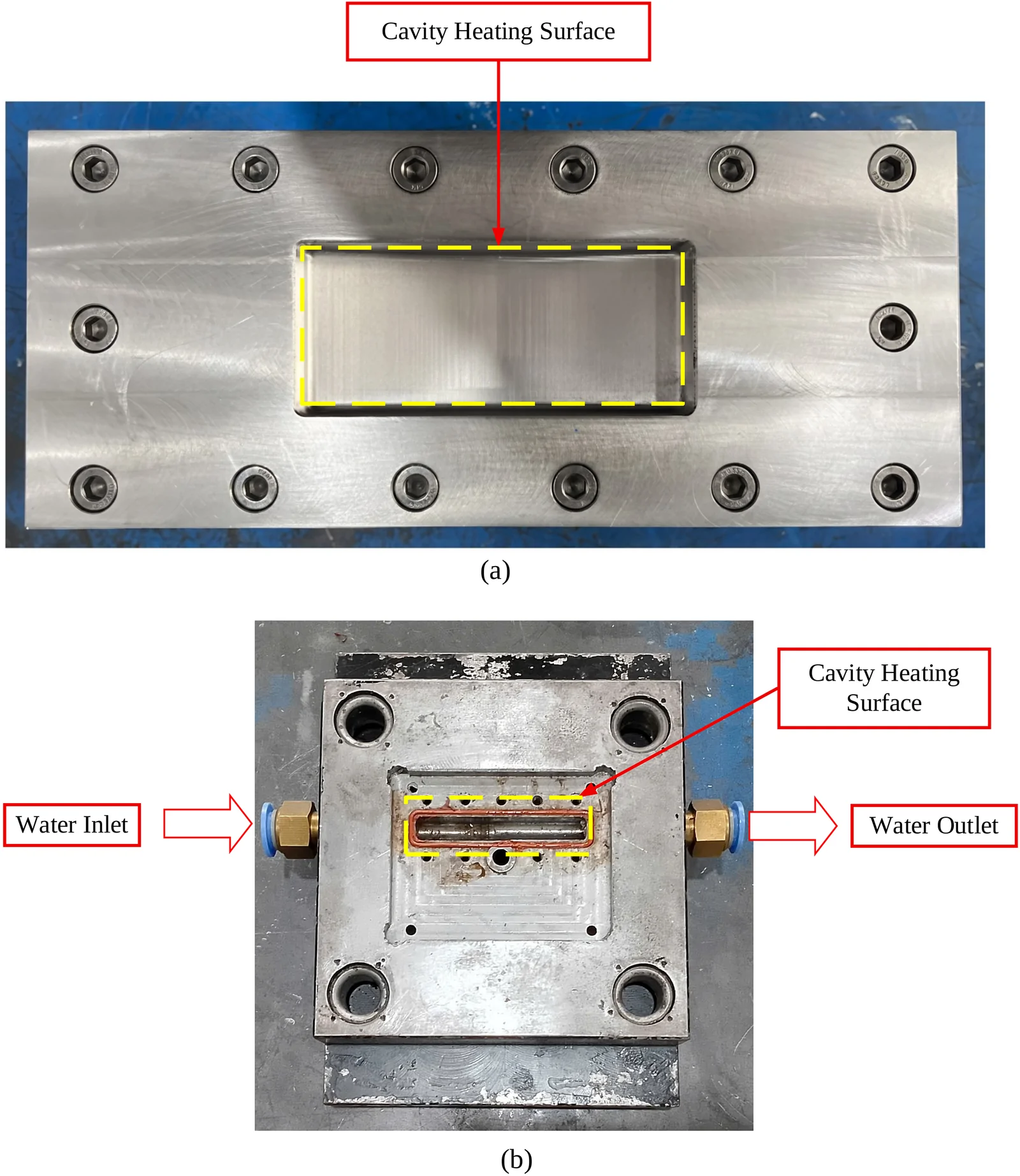
Actual fabricated components of the insert and cooling plates after precision machining: (a) Design 1; (b) Design 2. (All photographs were taken by the authors in their laboratory.).

The mold insert plate serves as the direct interface with the molten polymer and acts as the primary heated zone of the mold. Immediately beneath the insert, the cooling plate is CNC-milled to form fluid channels with a concave profile that closely conforms to the geometry of the upper mold cavity. The structural differences between the two designs are clearly characterized by the fluid channel depth and the thickness of the metallic insert plate, establishing contrasting heat transfer conditions (a 3 mm fluid layer in Design 1 and a 10 mm fluid layer in Design 2). This contoured design shortens the local heat-transfer path between the circulating fluid and the cavity surface, enabling heat to be supplied during the preheating stage and extracted through the same fluid layer during the subsequent cooling stage.

From a heat transfer perspective, system performance is governed not only by the geometry of the cooling layer but is also heavily influenced by the thermal contact conditions at the metallic interface. Therefore, the insert plate and cooling plate are assembled and securely fastened using a system of bolts evenly distributed along the perimeter to maintain stable contact pressure over multiple injection molding cycles. Minimizing the interfacial gap effectively reduces the thermal contact resistance and ensures mechanical rigidity under combined thermal loads and injection pressures. The internal cooling channels within the cooling plate are connected externally via fluid connectors, facilitating flexible integration with auxiliary temperature control systems. In actual operation, the modular insert–cooling plate assembly is installed such that the insert surface aligns with the cavity region of the injection mold. The circulating medium system is first activated to establish a baseline thermal state, after which a controlled heating process raises the cavity surface temperature to the target value. Once a relatively stable thermal state is achieved within the insert and the cooling layer, the injection molding cycle is initiated. Under this configuration, the temperature evolution of the cavity surface is governed by the coupled effects of active heating and forced cooling, faithfully replicating the actual operating conditions of thermally controlled injection molds. Overall, presenting the actual fabricated components (Fig 9) not only confirms the manufacturability of the fluid-layer mold structure via conventional machining methods, but also provides a solid physical foundation to investigate the temperature distribution and thermal response rate of the system.

#### 2.5.5. HDPE preparation and injection molding conditions.

High-density polyethylene (HDPE) was selected for evaluating the displacement response of the injection-molded bridge-type compliant mechanisms. Virgin and recycled HDPE were blended at recycled-material fractions of 0, 5, 10, 15, 20, and 25 wt%. The recycled fraction was defined on a mass basis. For each 800 g batch, the masses of virgin/recycled HDPE were 800/0, 760/40, 720/80, 680/120, 640/160, and 600/200 g, respectively, for recycled-HDPE contents of 0, 5, 10, 15, 20, and 25 wt%.

The injection-molding parameters were maintained constant for all HDPE blend ratios to isolate the effect of recycled-material content. The machine-controller filling-pressure setting was 26 bar, the filling-speed setting was 65%, and the filling time was 2 s. The machine-controller packing-pressure setting was 23 bar, with a packing-speed setting of 60% and a packing time of 1 s. The barrel-temperature setting was maintained within the range of 200–210 °C. These pressure values are controller settings recorded during molding and should not be interpreted as direct nozzle or cavity melt-pressure measurements. The same molding settings were applied to all recycled-HDPE ratios.

#### 2.5.6. Displacement measurement of the compliant mechanisms.

The displacement response of the molded bridge-type compliant mechanisms was evaluated using a custom mechanical fixture. Input displacement was applied using a screw-driven loading mechanism acting on the two input blocks of the mechanism. Two dial indicators were positioned symmetrically at the input locations, while the output displacement was measured at the output block.

*Measurement schematic: [Input indicator 1] → [left input block] — bridge-type compliant mechanism — [right input block] ← [Input indicator 2]; [Output indicator] measures the displacement of the central output block while the screw-driven fixture imposes the input displacement.*

Before each test, the indicators were zeroed after the specimen had been mounted and aligned in the fixture. The screw-driven mechanism was then incrementally advanced to impose increasing input displacement. The investigated displacement range was divided into approximately ten increasing levels between the initial position and the maximum displacement considered for the specimen. At each loading position, the input displacement was calculated as the average of the two measurements obtained from the two input sides.

For each recycled-HDPE fraction, three molded specimens were randomly selected for displacement testing. The output displacement reported at each input level represents the mean value obtained from the three specimens. The displacement response was evaluated over the recycled-HDPE range from 0 to 25 wt%.

## 3. Results and discussion

### 3.1. Validation of thermo–fluid simulation model

To establish the reliability of the numerical model prior to conducting benchmark analyses and parameter optimization, the transient heat transfer process on the cavity surface was cross-validated against experimental measurements. The validation process was performed comprehensively through both qualitative assessment (thermal map visualization) and quantitative evaluation (localized temperature error) under two distinct operating designs.

First, the spatial similarity of the temperature fields was visually compared. Fig 10 presents a comparison between infrared thermal images from the experiment and simulated temperature contours for Design 1 (insert thickness 1 mm, fluid layer thickness 3 mm, inlet temperature 60 °C).

**Fig 10:**
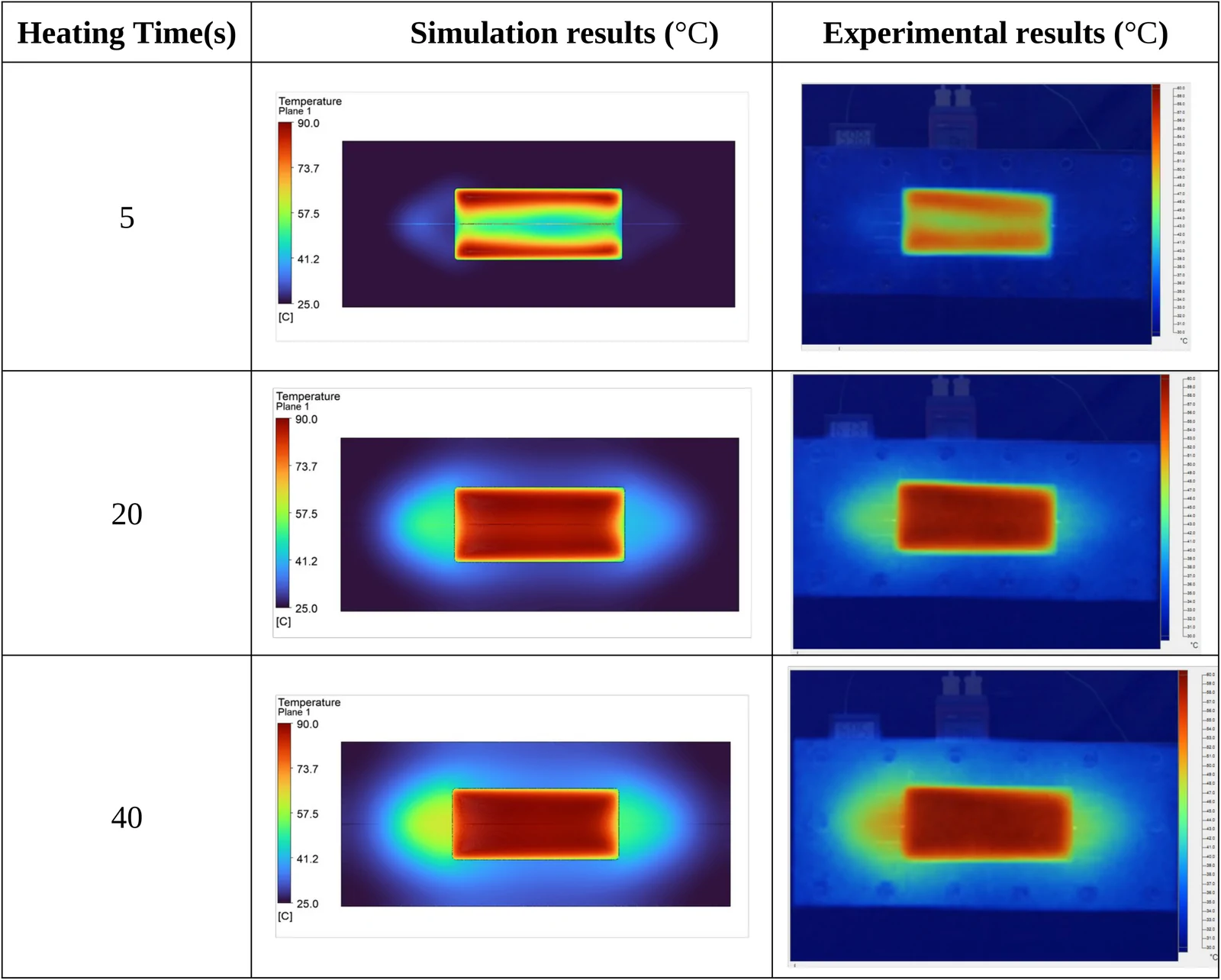
Simulated and experimental mold heating responses for a 1 mm insert with a 3 mm water layer at an inlet temperature of 60 °C and a cooling water flow rate of 51 g/s. (Experimental thermograms were acquired by the authors, and the corresponding temperature contours were generated from the authors’ numerical simulations).

To further verify the model’s response limits under higher-intensity heat transfer conditions, Fig 11 illustrates the thermal distribution for Design 2 (insert thickness reduced to 0.5 mm, fluid layer thickness 10 mm, inlet temperature 80 °C). In both cases, the simulated and experimental fields showed similar overall spatial heating patterns: high temperatures concentrated at the center of the cavity, forming symmetrical thermal gradient bands spreading outwards toward the parting surface edges.

**Fig 11:**
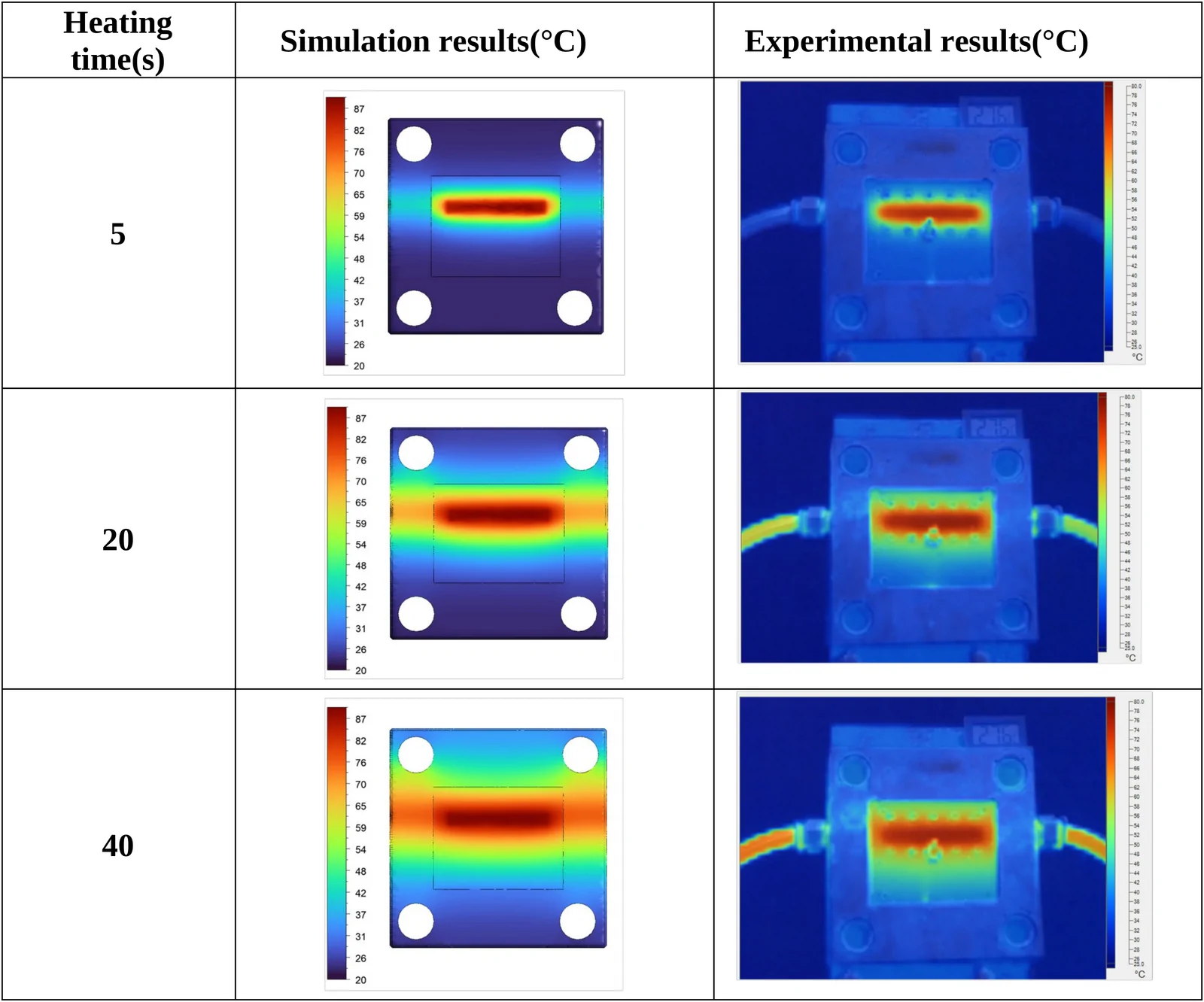
Simulated and experimental mold heating responses for a 0.5 mm insert with a 10.

mm water layer at an inlet temperature of 80 °C and a water flow rate of 59.5 g/s (Experimental thermograms were acquired by the authors, and the corresponding temperature contours were generated from the authors’ numerical simulations)

Although the simulated and experimental temperature fields showed similar overall spatial trends, a quantitative comparison was performed to evaluate the prediction error. Table 2 compiles the temperature values extracted at Point P (for Design 1) and at the center of the measurement line (for Design 2, which is geometrically identical to Point P) to clarify the absolute deviation at each time step.

**Table 2:** Simulated and experimental temperature responses for model validation.

Operating condition	Heating time (s)	Simulation results (°C)	Experimental results (°C)	Absolute error(°C)
Design 1	5	36.41	50.30	13.89
	20	53.34	58.00	4.66
	40	55.70	58.70	3.00
Design 2	5	77.86	74.70	3.16
	20	79.46	77.40	2.06
	40	79.61	76.30	3.31

Statistical metrics were calculated based on the dataset in Table 2 to accurately assess the model’s reliability. For the six validation points in Table 2, the Mean Absolute Error (MAE) and Root Mean Square Error (RMSE) were 5.01 °C and 6.44 °C, respectively. The temporal error distribution shows that the largest discrepancy occurred during the early transient stage. The maximum deviation (13.89 °C) occurred at 5 s for Design 1, when the experimental temperature exceeded the simulated value. Because the sign of this deviation is inconsistent with a simple physical thermal-lag explanation, it is more appropriately attributed to uncertainties in the initial and boundary conditions, thermal contact conditions, and infrared measurement during the early transient period. For Design 2, the absolute error at 5 s was smaller (3.16 °C), and the simulated and experimental temperatures remained within approximately 2–3 °C at 20 and 40 s. The lower error in Design 2 reflects the combined effects of the smaller insert thickness, larger fluid-layer depth, higher inlet temperature, and higher flow rate. Because these parameters changed simultaneously, the contribution of insert thickness alone cannot be isolated from the two validation cases.

Fig 12 presents the simulated 3D cavity-surface temperature distributions for Design 1 at 5, 20, and 40 s. At 5 s (Fig 12a), heating remains concentrated near the central molding region and the surface exhibits comparatively steep spatial gradients. The relatively large simulation–experiment discrepancy at this time (13.89 °C) should be interpreted together with the early-transient measurement and boundary-condition uncertainties discussed above. At 20 s (Fig 12b), the simulated central temperature increases to 53.34 °C and the high-temperature region expands. At 40 s (Fig 12c), the surface approaches a quasi-steady thermal condition, with a simulated central temperature of 55.70 °C compared with 58.70 °C experimentally. The transition from a localized early hot region to a broader temperature plateau indicates progressive spatial homogenization before injection, although a finite surface-temperature gradient remains.

**Fig 12:**
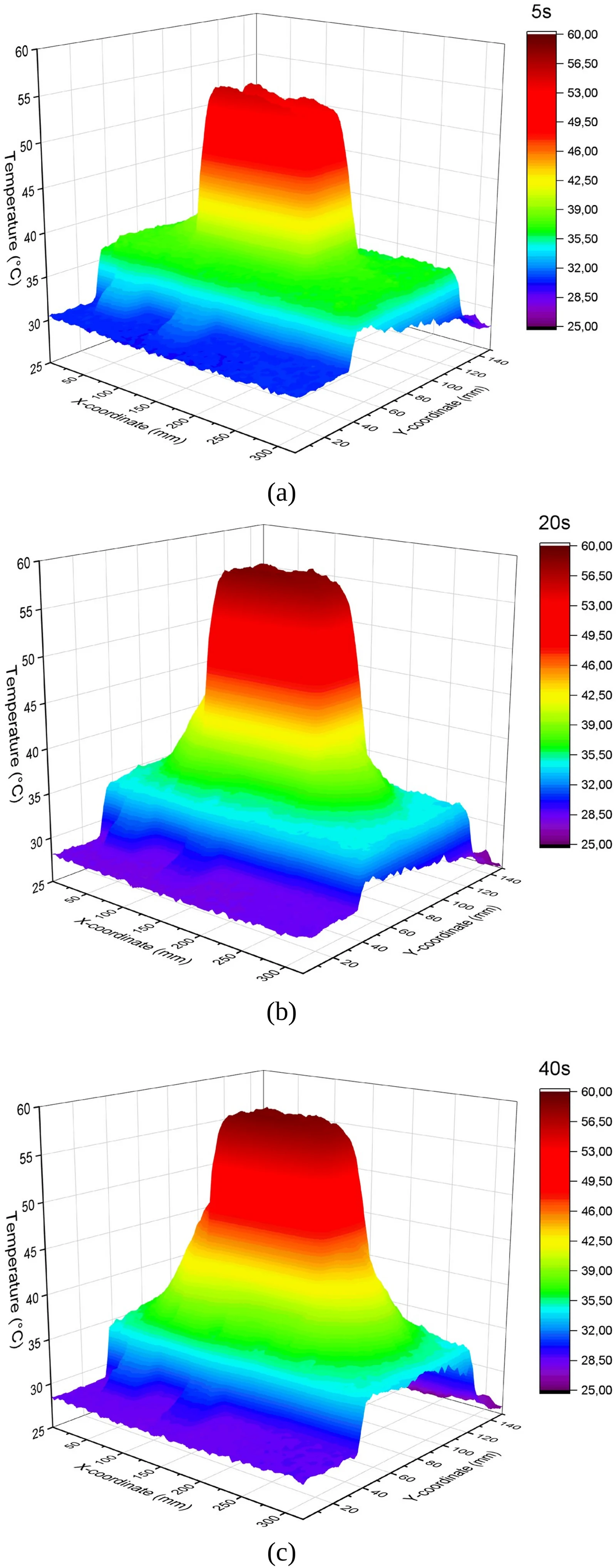
3D spatiotemporal temperature topographies of the mold surface for Design 1 at different heating intervals: (a) t = 5 s; (b) t = 20 s; (c) t = 40 s.

### 3.2. Experimental and numerical comparison between straight-channel and fluid-layer mold temperature control

The fluid-layer architecture was compared with a conventional straight-channel configuration using two complementary levels of evidence. First, a numerical benchmark was performed under identical prescribed operating conditions (80 °C inlet temperature, 59.5 g/s mass flow rate, and 40 s heating) to isolate the effect of heat-transfer architecture. Second, archived radiometric thermography from a representative steel straight-channel insert operated at the same inlet temperature and mass flow rate was reprocessed and compared with the existing steel fluid-layer measurements. The experimental comparison is used as an independent directional thermal benchmark rather than as a one-to-one validation of the exact numerical straight-channel geometry. Fig 13 first presents the numerical temperature contours of the two architectures.

**Fig 13:**
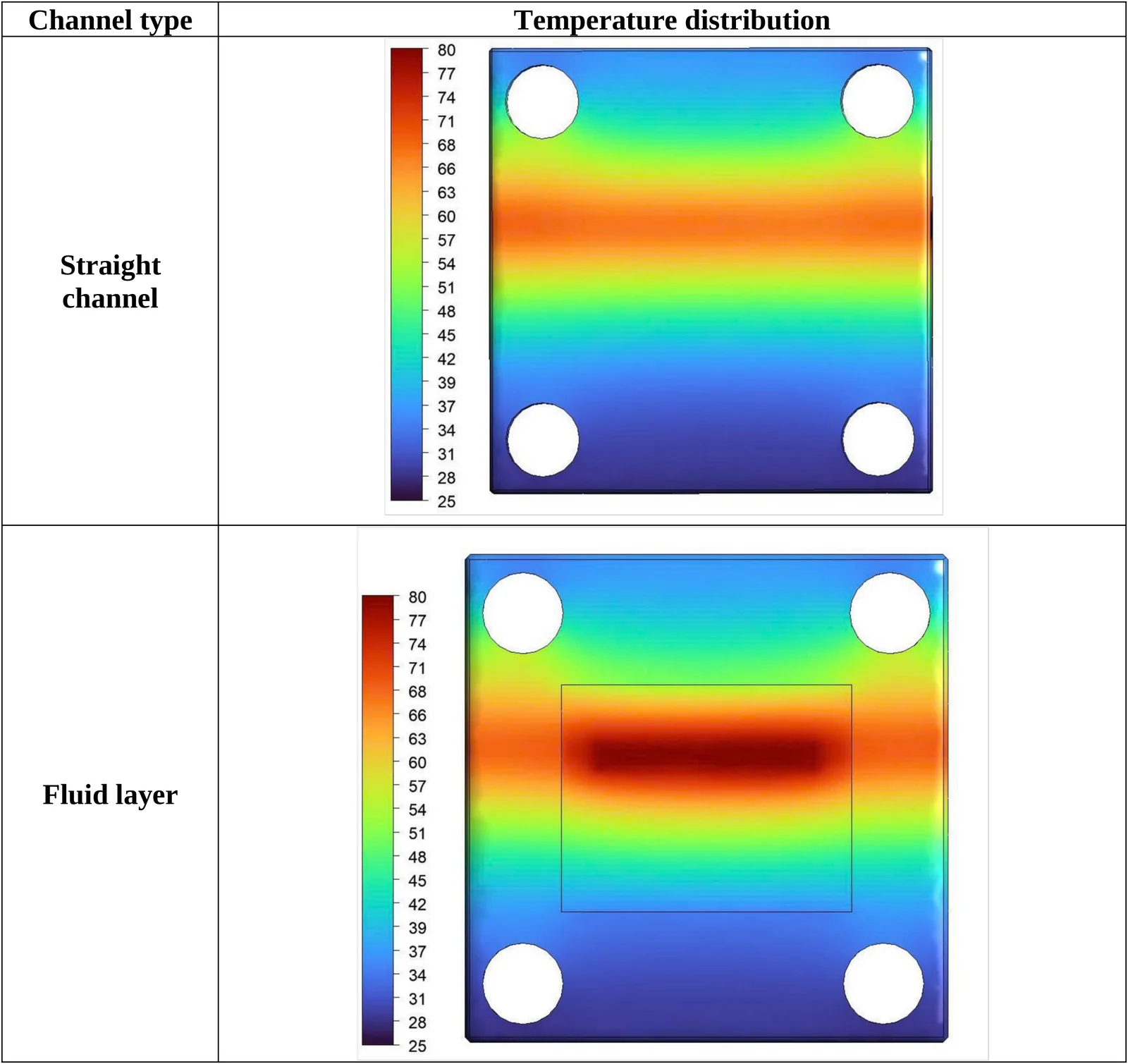
Comparison of simulated temperature contours between the conventional straight-channel design and the proposed fluid-layer architecture under identical operating conditions.

The difference in early transient heat distribution is illustrated by the simulated 3D temperature maps at 5 s (Fig 14). The straight-channel architecture exhibits localized heating above the discrete channels and lower-temperature regions between them, whereas the fluid-layer architecture develops a broader high-temperature region over the molding zone. The numerical benchmark at 40 s predicted an average cavity-surface temperature of approximately 76 °C for the straight-channel configuration and approximately 80 °C for the fluid-layer configuration. The corresponding numerical maximum longitudinal temperature difference decreased from approximately 14–10 °C, equivalent to a 28.6% reduction. These numerical metrics are retained as simulation results. The experimental straight-channel benchmark added in this revision is evaluated separately using the registered center point and longitudinal measurement line, as summarized in Table 3 and Fig 15 and Fig 16.

**Fig 14:**
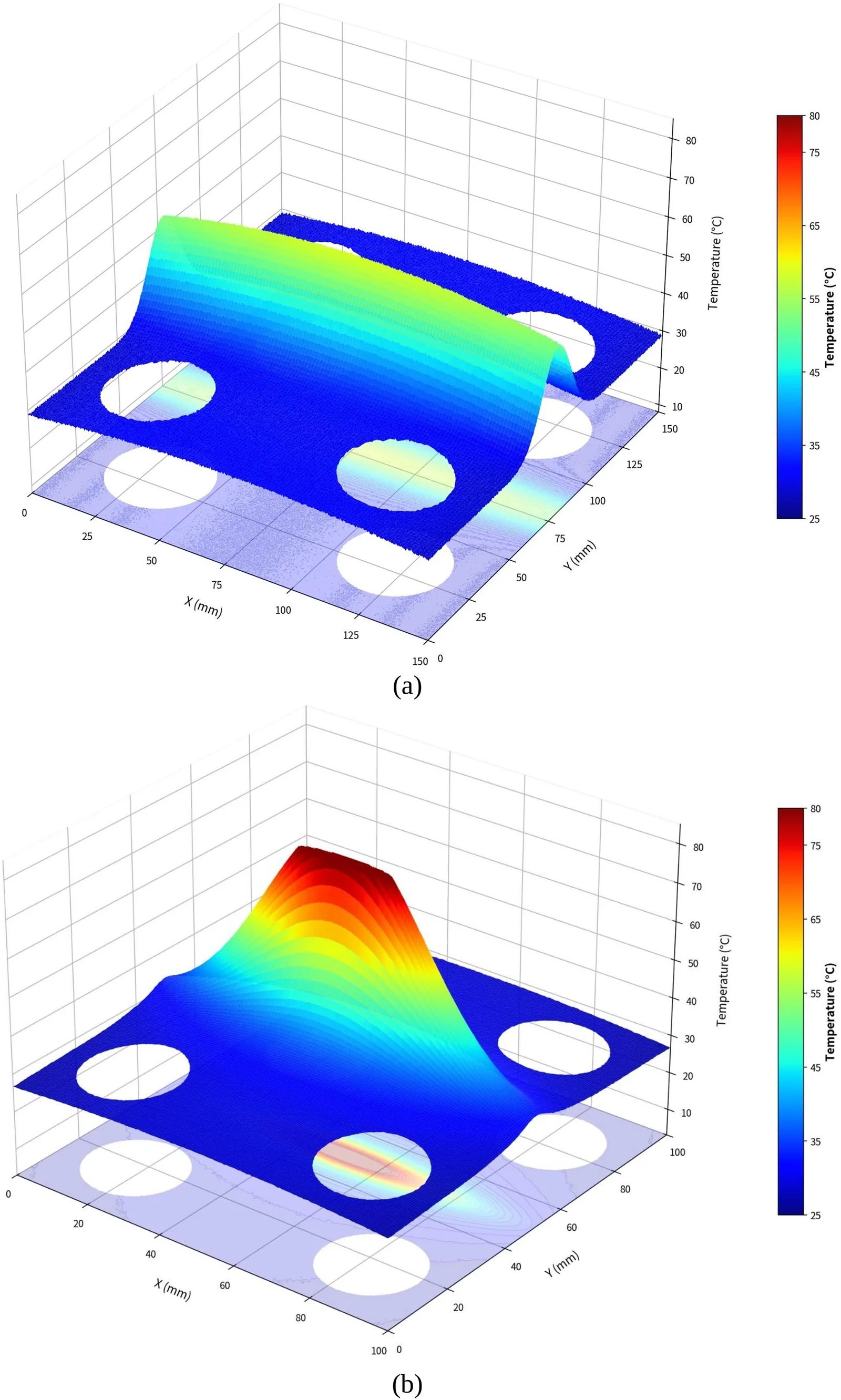
3D spatiotemporal temperature topographies of the mold surface at t = 5 s under identical operating conditions (Inlet: 80 °C, Flow rate: 59.5 g/s): (a) Conventional straight-channel architecture; (b) Proposed fluid-layer architecture.

**Table 3:** Combined numerical and representative experimental thermal benchmark of the conventional straight-channel and proposed fluid-layer architectures and proposed fluid-layer architecture (Time: 40 s, Inlet: 80 °C).

Metric	Straight channel	Fluid layer	Evidence/ interpretation
Numerical average cavity-surface temperature at 40 s	~76 °C	~80 °C	Simulation; + 4.0 °C for fluid layer
Numerical maximum longitudinal temperature difference at 40 s	~14 °C	~10 °C	Simulation; 28.6% reduction
Experimental central cavity-surface temperature at 40 s	59.9 °C	76.3 °C	Representative thermography; same 80 °C inlet and 59.5 g/s flow
Experimental longitudinal line mean at 40 s	57.9 °C	75.6 °C	Line-based experimental comparison
Experimental longitudinal line range, ΔTline, at 40 s	12.4 °C	1.5 °C	Representative line-based non-uniformity; not identical to the area-based numerical metric
Melt-filling behavior	Incomplete filling predicted	Complete filling predicted	Simulation only; no new straight-channel molding experiment

**Fig 15:**
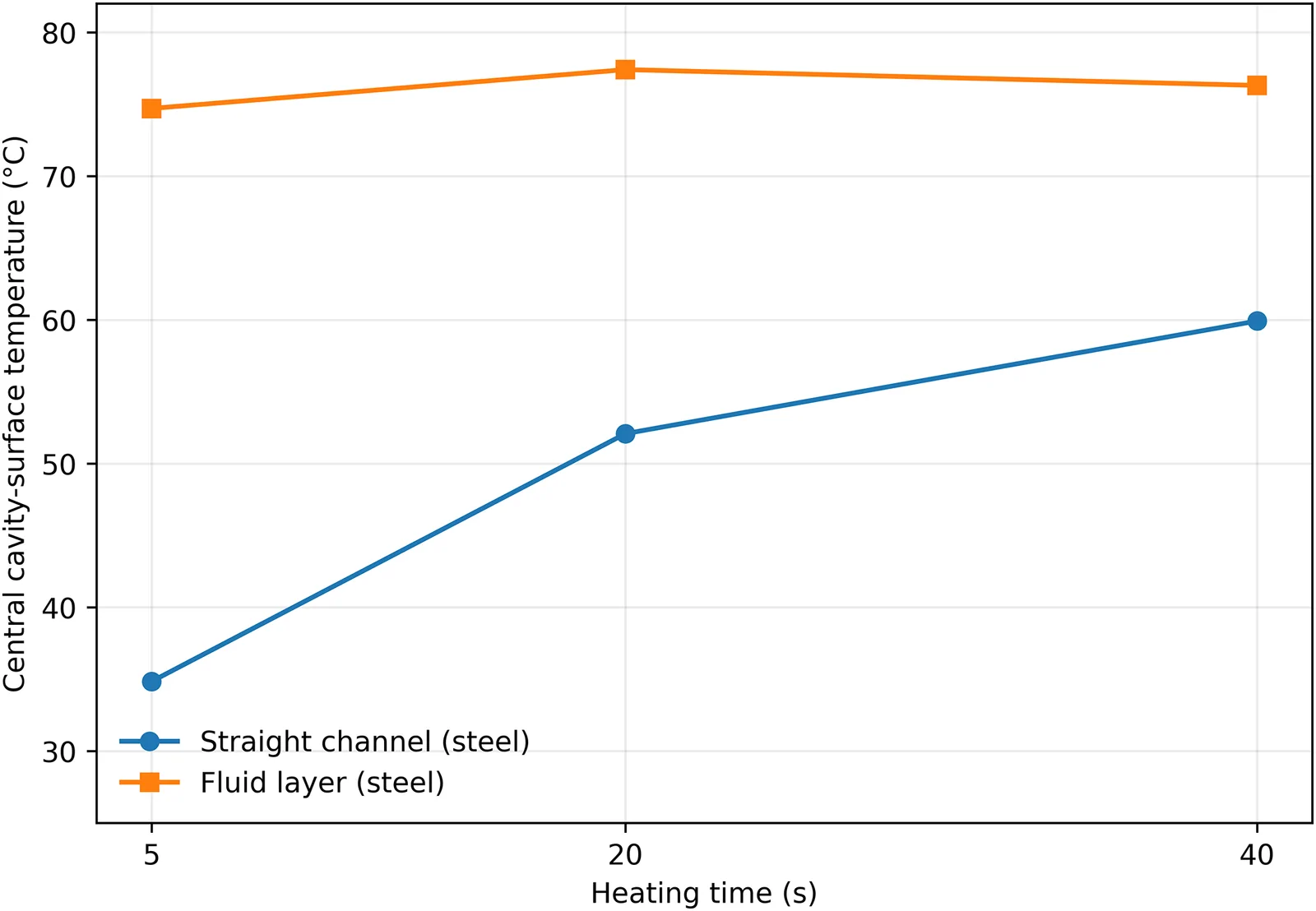
Representative experimental central cavity-surface temperature responses of the steel straight-channel and steel fluid-layer configurations under an inlet-water temperature of 80 °C and a mass flow rate of 59.5 g/s.

**Fig 16:**
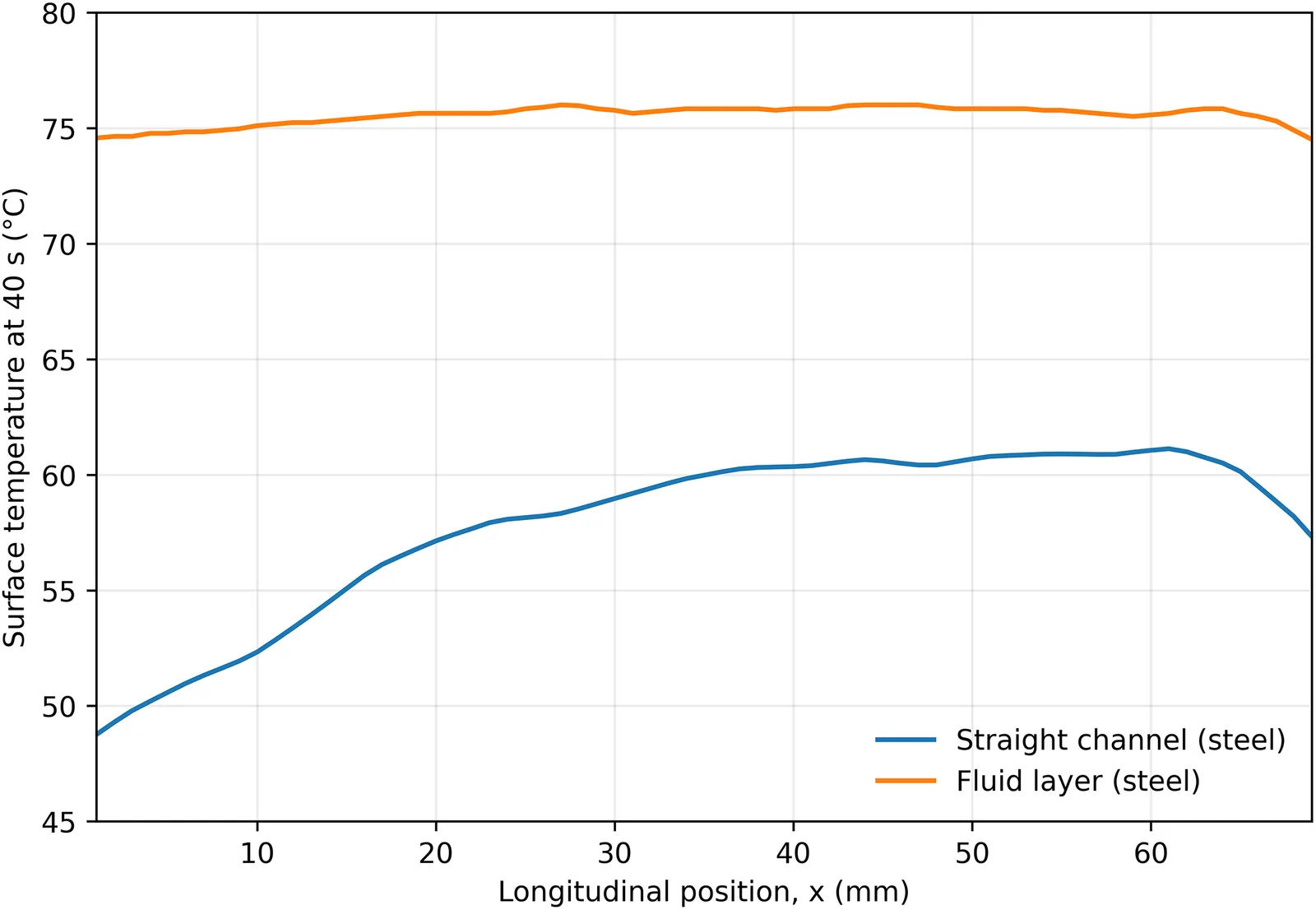
Representative experimental longitudinal cavity-surface temperature profiles at 40 s for the steel straight-channel and steel fluid-layer configurations under an inlet-water temperature of 80 °C and a mass flow rate of 59.5 g/s.

The combined benchmark shows that the numerical and experimental evidence point in the same direction: the fluid-layer architecture develops a higher and more spatially uniform cavity-surface temperature than the conventional straight-channel baseline. In the numerical benchmark, the predicted differences were +4.0 °C in average surface temperature and −28.6% in the maximum longitudinal temperature difference. In the representative thermography experiment, the central surface temperature at 40 s was 59.9 °C for the straight-channel insert and 76.3 °C for the fluid-layer insert. The corresponding longitudinal line profiles were also markedly different: the line mean was 57.9 °C for the straight-channel insert and 75.6 °C for the fluid-layer insert, while the line temperature range was 12.4 °C and 1.5 °C, respectively. Because the experimental straight-channel geometry is a representative conventional baseline rather than an exact replica of the numerical geometry, the experimental values are not used to validate the numerical +4.0 °C and 28.6% magnitudes one-to-one. Instead, they provide independent experimental confirmation of the direction of the thermal advantage. No experimental straight-channel molding was performed; therefore, the subsequent short-shot/complete-filling comparison remains simulation-based.

The straight-channel configuration exhibited larger simulated temperature decreases toward the peripheral regions, indicating greater spatial non-uniformity for the evaluated geometry. The melt-filling analysis subsequently predicted an incomplete flexure-hinge region for this numerical straight-channel case. Because no new straight-channel injection-molding experiment was performed in this revision, this filling result is treated as a simulation prediction and is not used as experimental evidence of mechanical-test readiness.

Fig 15 shows the experimental center-point response used to complement the numerical benchmark. The fluid-layer configuration reached approximately 74.7 °C by 5 s and remained close to the imposed thermal level through 40 s, whereas the straight-channel steel insert increased more gradually from approximately 34.8 °C at 5 s to 59.9 °C at 40 s. The comparison demonstrates a substantially faster experimental thermal response for the fluid-layer configuration under the investigated setup. Because the straight-channel record is a representative archived sequence rather than a repeated test series, no error bars are assigned to this comparison.

The 40-s profiles in Fig 16 further demonstrate the difference in spatial uniformity. Across the registered longitudinal molding span, the straight-channel profile had a line mean of approximately 57.9 °C and a line temperature range of approximately 12.4 °C, whereas the fluid-layer profile had a mean of approximately 75.6 °C and a range of approximately 1.5 °C. These line-based experimental values are not numerically identical to the area-based ΔTmax metric used in the numerical benchmark, but they provide independent evidence that the fluid-layer configuration generated a flatter temperature profile over the measured molding region.

### 3.3. Parametric effect of fluid-layer thickness

Following the benchmark comparison, the effects of operating and geometric parameters on the thermal response of the fluid-layer architecture were evaluated. The transient cavity-surface temperature was evaluated for geometric parameter values t = 3, 5, and 8 mm and inlet temperatures of 60 and 80 °C. The temporal temperature evolution at the central measurement point (Point P) for different thickness designs was investigated in detail. Fig 17–18, and Fig 19 illustrate the thermal response of Design 1 (insert 1 mm) combined with fluid layer thicknesses of 3 mm, 5 mm, and 8 mm, respectively, under the same 60 °C inlet condition. The data shows that across all designs, the cavity surface temperature increases rapidly within the first 15–20 seconds; thereafter, the heating rate decelerates as the system approaches a quasi-steady thermal condition. Notably, the thinnest fluid layer design (t = 3 mm) exhibited the fastest thermal response rate and achieved the highest surface temperature at the end of the heating cycle (40 s). As the fluid-layer thickness increased from 3 mm to 5 and 8 mm, the slope of the temperature curve decreased, and the peak achievable temperature was lower.

**Fig 17:**
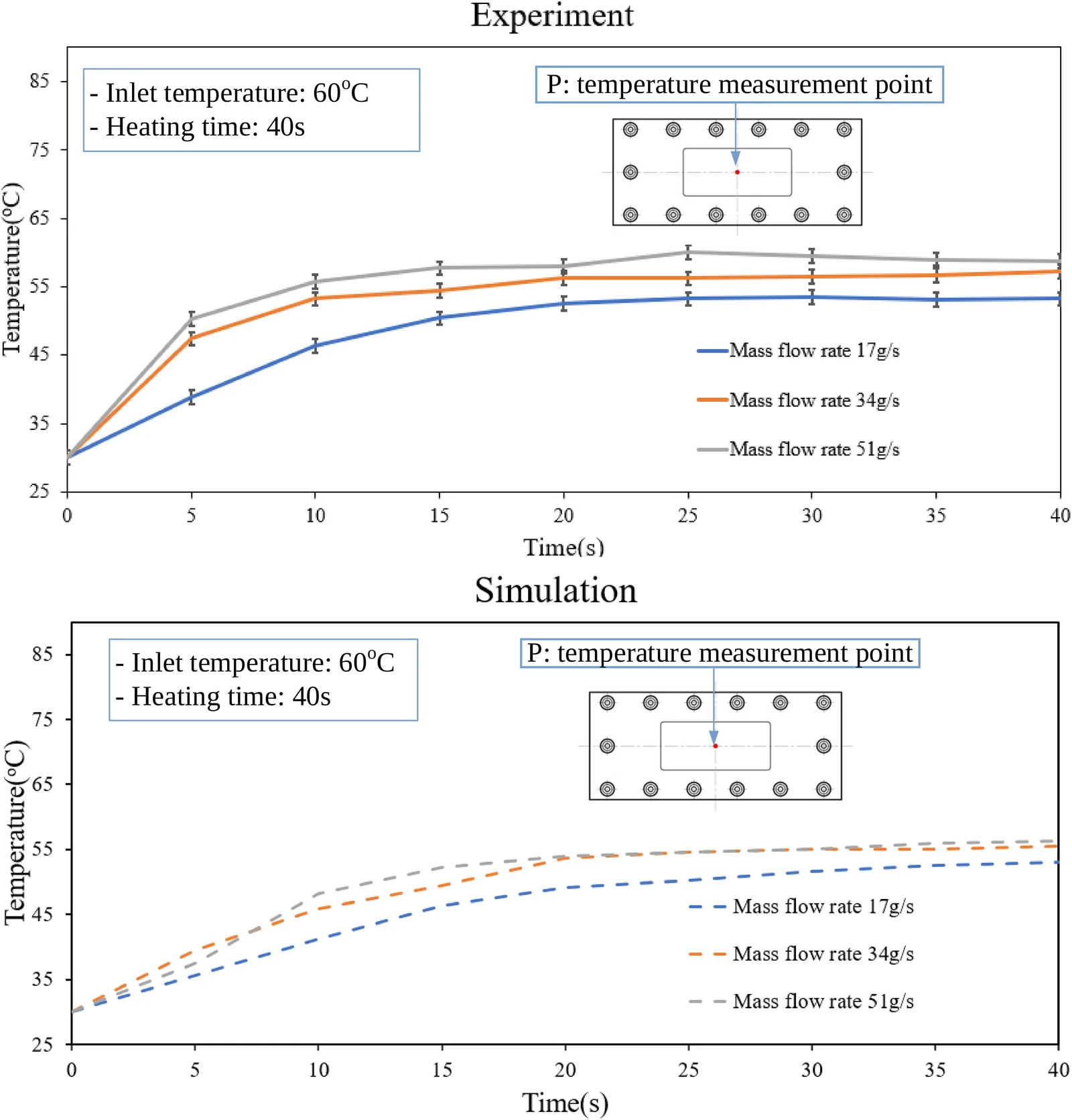
Experimental and simulated transient temperatures at point P for the 1 mm insert and 3 mm water layer at an inlet temperature of 60 °C under different cooling water flow rates.

**Fig 18:**
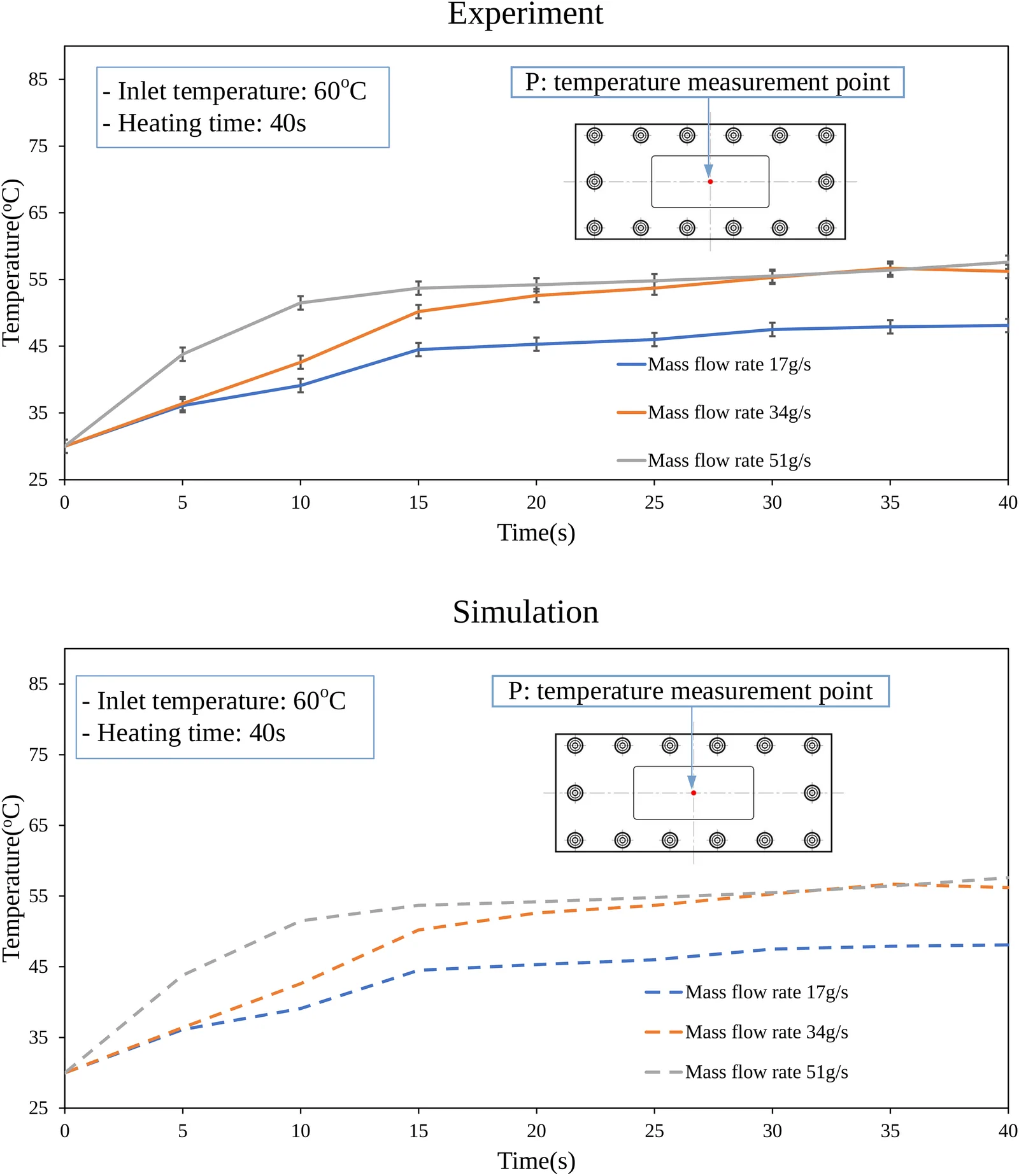
Experimental and simulated transient temperatures at point P for the 1 mm insert and 5 mm water layer at an inlet temperature of 60 °C under different cooling water flow rates.

**Fig 19:**
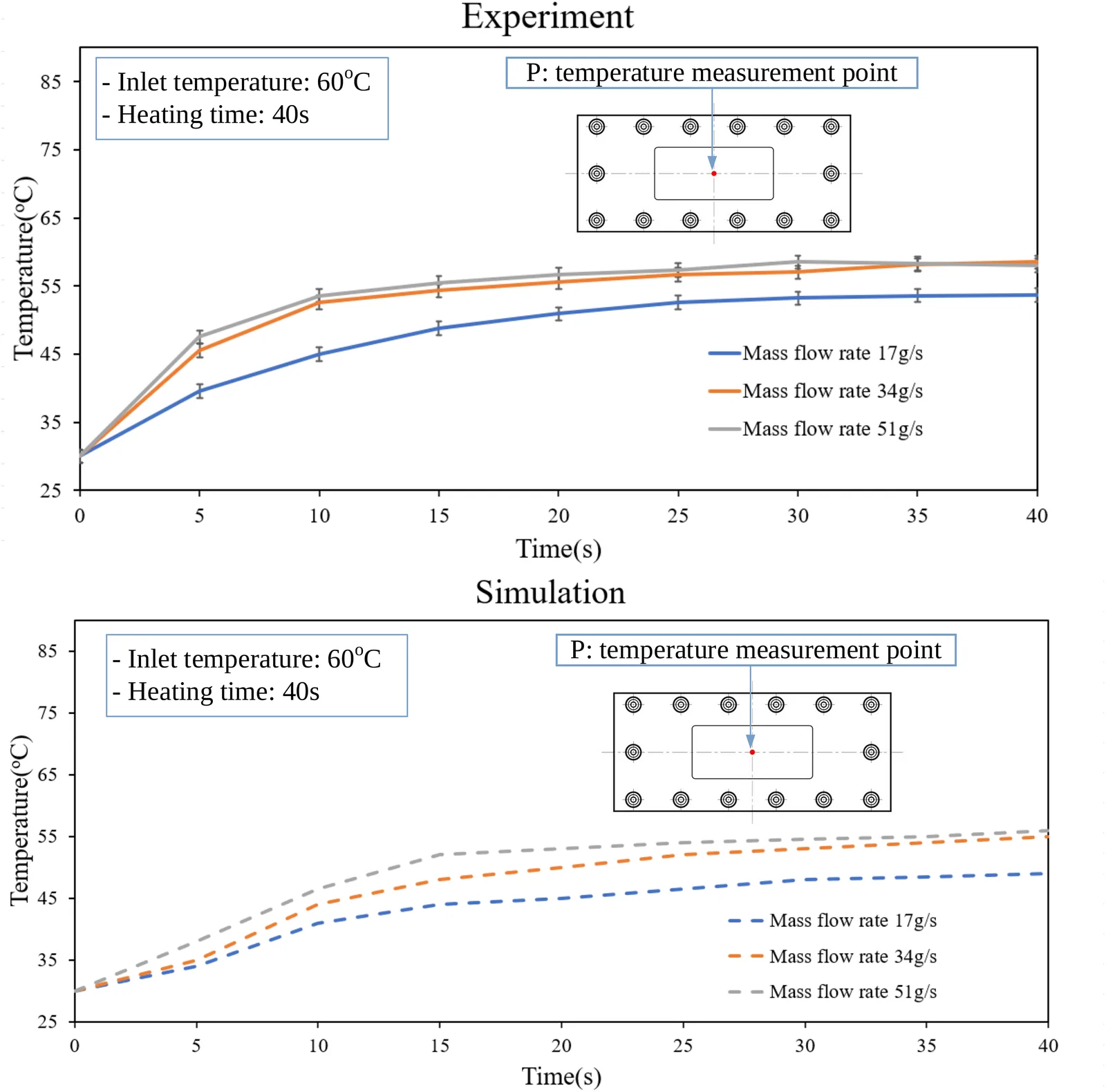
Experimental and simulated transient temperatures at point P for the 1 mm insert and 8 mm water layer at an inlet temperature of 60 °C under different cooling water flow rates.

The influence of the inlet temperature is more pronounced in Fig 20 and Fig 21, corresponding to the 3 mm and 5 mm fluid layer designs when the supply temperature is elevated to 80 °C. Compared to the 60 °C cases, the temperature curves shift upward, indicating a higher thermal response. However, the qualitative thickness trend remains similar: a thinner fluid-layer structure consistently yields a faster heating rate and a higher absolute surface temperature.

**Fig 20:**
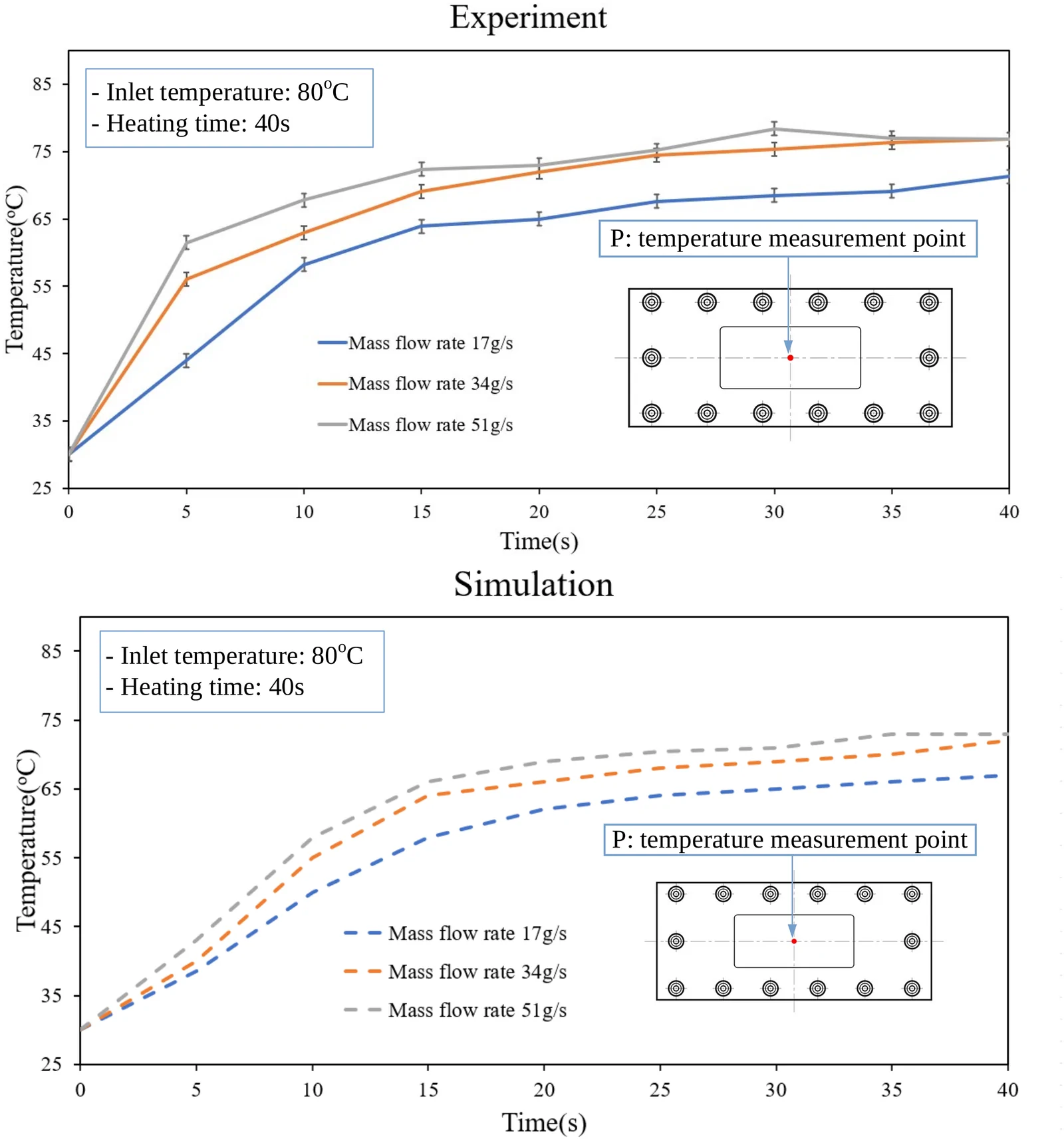
Experimental and simulated transient temperatures at point P for the 1 mm insert and 3 mm water layer at an inlet temperature of 80 °C under different cooling water flow rates.

**Fig 21:**
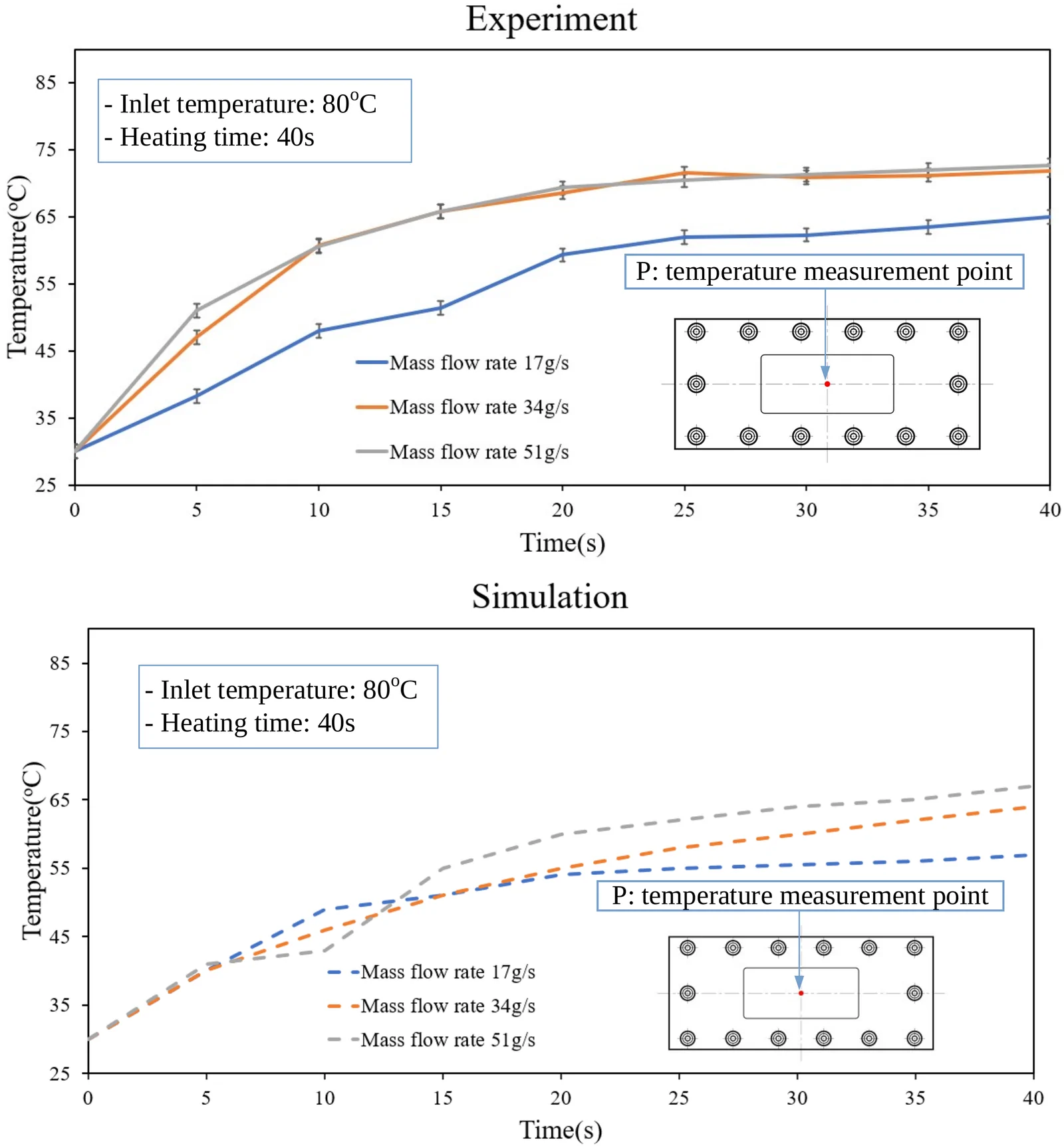
Experimental and simulated transient temperatures at point P for the 1 mm insert and 5 mm water layer at an inlet temperature of 80 °C under different cooling water flow rates. The experimental data and numerical results were generated by the authors.

The effects of fluid-layer thickness and insert thickness should be distinguished. The insert thickness primarily determines the solid conduction resistance between the circulating fluid and the cavity surface. In contrast, changing the fluid-layer thickness at a fixed mass flow rate changes the flow cross-sectional area, mean velocity, hydraulic conditions, and the resulting convective transport. Therefore, the faster thermal response observed for the 3 mm fluid layer should be interpreted as the combined result of fluid-dynamic and convective heat-transfer conditions rather than solely as a reduction in the solid conduction distance. Increasing the inlet temperature from 60 to 80 °C increases the temperature difference between the circulating fluid and the mold and therefore increases the thermal driving force, without changing this distinction between fluid-layer and insert-thickness effects.

Fig 22 shows that a longitudinal cavity-surface temperature gradient was present during the early heating stage at 5 s. As heating progressed, the temperature profile became progressively flatter, and the longitudinal temperature difference decreased substantially at 20 and 40 s. For this configuration, the 0.5 mm insert provides a short solid conduction path, while the 10 mm fluid layer and imposed mass flow rate determine the associated hydraulic and convective conditions. The combined thermal and flow conditions therefore produced a progressively more uniform cavity-surface temperature distribution during the investigated heating period.

**Fig 22:**
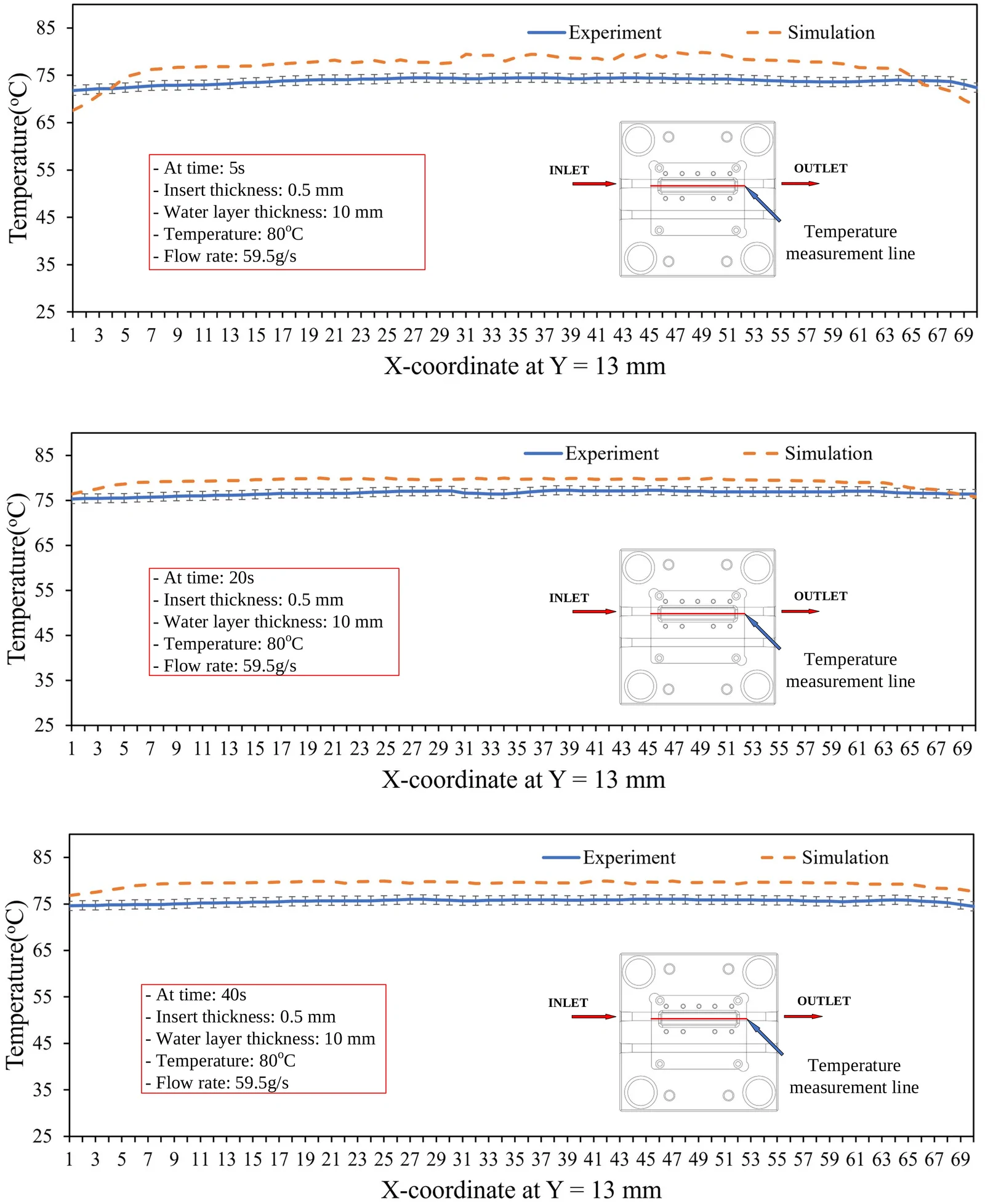
Experimental and simulated transient temperature profiles along the measurement line for a 0.5 mm insert with a 10 mm water layer at an inlet temperature of 80 °C and a flow rate of 59.5 g/s. The experimental data and numerical results were generated by the authors.

Taken together, the results indicate that a short local heat-transfer path combined with appropriate flow conditions can establish a higher and more uniform cavity temperature. These conditions can reduce the likelihood of premature melt solidification and improve mold filling in thin sections. For thin-walled compliant mechanisms, a more uniform cavity temperature may reduce asymmetric thermal histories across the flexure regions. The corresponding displacement response is evaluated in the next section.

### 3.4. Simulated mold filling and experimental compliant-mechanism displacement response

The surface temperature field control capability of the cooling system must be evaluated through the replication quality of the actual injection-molded parts. For micro-mechanical components with complex, thin-walled structures, the characteristics of the mold wall temperature distribution strongly influence the melt flow fillability and manufacturing tolerances. As previously detailed in the geometric analysis in Fig 8, the 1 mm flexure hinges of the bridge-type mechanism function as high-resistance hydrodynamic bottlenecks, causing the molten polymer to undergo sudden heat loss and localized viscosity spikes. This filling challenge may become more pronounced when recycled material is introduced because repeated processing can alter the rheological and thermal behavior of the polymer. To clarify the influence of thermal conditions in overcoming this “bottleneck”, the transient flow dynamics of the polymer are isolated and analyzed in detail in Fig 23.

**Fig 23:**
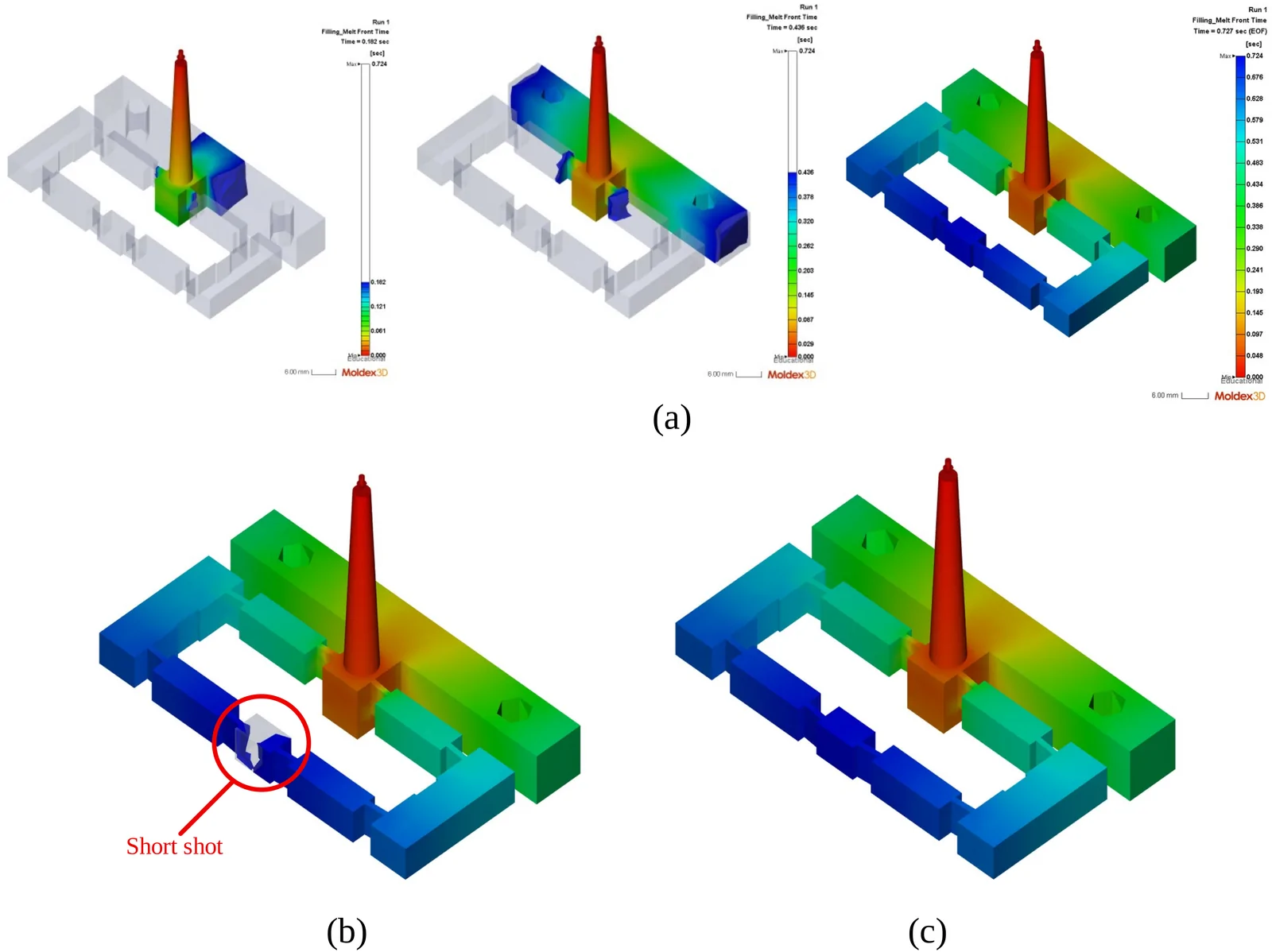
Simulated melt-filling behavior of the bridge-type compliant mechanism: (a) melt-front progression through three filling stages; (b) predicted incomplete filling for the conventional straight-channel configuration; and (c) predicted complete filling for the proposed fluid-layer configuration.

Fig 23a details the kinematics of the melt front progression, divided into 3 core filling stages. In Stage 1 (initial filling), the molten polymer originates from the central sprue and propagates symmetrically into the base block. Entering Stage 2 (passing the bottleneck), the flow advances into the flexure hinges; here, the abruptly narrowed mold wall cross-section subjects the polymer melt to substantial flow resistance, as indicated by the deceleration of the melt front. Finally, in Stage 3 (filling completion), the melt front exits the hinges and continues to expand, filling the entire molding cavity up to the furthest peripheral edges. Isolating this 3-stage sequence highlights the natural rheological behavior of the polymer, establishing a basis for evaluating the flow sensitivity to the mold wall thermal conditions of each cooling design. For the conventional straight-channel configuration, Fig 23b shows an incomplete-filling region at the flexure hinge. The predicted melt front did not pass completely through the flexure-hinge region in Stage 2, resulting in a short-shot region at the location indicated in Fig 23b. The lower local mold temperature predicted for the straight-channel case promotes faster solidification near the wall, which reduces the available flow area through the thin hinge region. Fig 23c predicts complete filling for the fluid-layer configuration under the investigated conditions. The higher and more uniform mold-surface temperature in the fluid-layer case reduced heat loss from the melt as it passed through the thin flexure region. The uniform mold wall temperature kept the melt viscosity at a lower level through the bottleneck, which is consistent with the complete-filling prediction under the investigated conditions. Alongside filling capability, the pressure distribution at the end of filling (EOF) was also extracted (Fig 24) to assess the hydrodynamic resistance of the mechanism.

**Fig 24:**
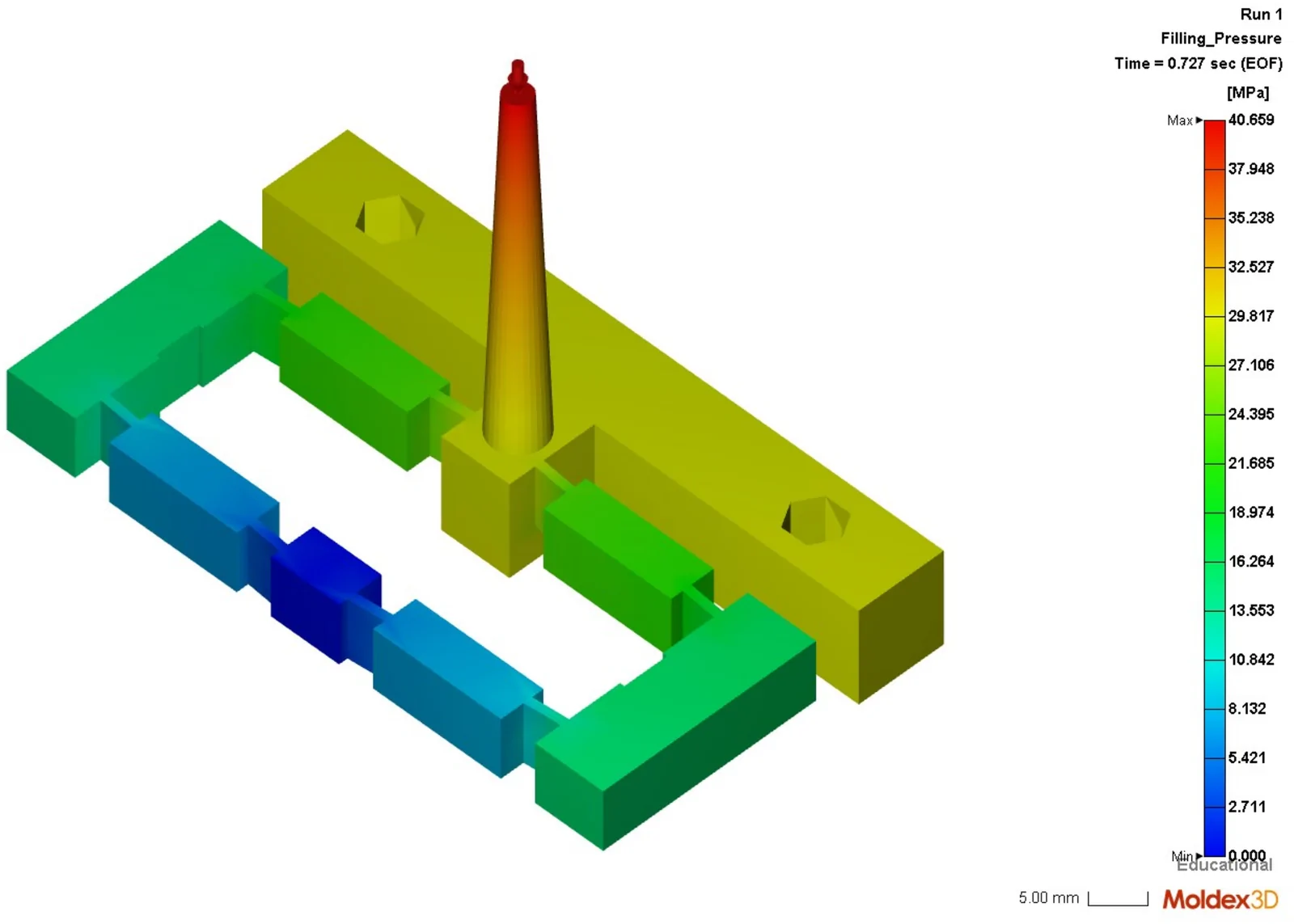
Pressure distribution at the end of filling (EOF) for the proposed fluid-layer mold architecture.

The melt-filling simulation predicted that the higher and more uniform wall-temperature field of the fluid-layer case delayed frozen-layer growth through the thin hinges. The predicted peak melt pressure at the end of filling was approximately 40.6 MPa, and the simulated pressure field decreased smoothly from the central feed region toward the cavity extremities. This 40.6 MPa value is a simulated melt-pressure result and is distinct from the machine-controller pressure settings reported in Table 4. These filling and pressure results are numerical and are not presented as experimental straight-channel molding validation.

**Table 4:** HDPE blend compositions and injection-molding conditions.

Parameter	Value
Recycled-HDPE fraction	0, 5, 10, 15, 20, 25 wt%
Total batch mass	800 g
Virgin/recycled HDPE at 0%	800/0 g
Virgin/recycled HDPE at 5%	760/40 g
Virgin/recycled HDPE at 10%	720/80 g
Virgin/recycled HDPE at 15%	680/120 g
Virgin/recycled HDPE at 20%	640/160 g
Virgin/recycled HDPE at 25%	600/200 g
Machine-controller filling-pressure setting	26 bar
Filling-speed setting	65%
Filling time	2 s
Machine-controller packing-pressure setting	23 bar
Packing-speed setting	60%
Packing time	1 s
Machine temperature setting	200–210 °C

Analysis of Fig 25 shows an approximately monotonic and near-linear relationship between input and output displacement for the HDPE compliant mechanisms over the investigated range. Each curve represents the mean response of three specimens, with the error bars indicating ±1 SD. As the recycled-HDPE fraction increased, the output displacement at a given input displacement generally decreased. At an input displacement of approximately 175 µm, the mean output displacement decreased from 857 µm for virgin HDPE to 745 µm for the blend containing 25 wt% recycled HDPE, corresponding to a reduction of approximately 13.1%. The endpoint displacement amplification ratio δout/δin therefore decreased from approximately 4.90 to 4.26. Linear fits to the mean input–output curves gave effective slopes of approximately 4.88, 4.74, 4.55, 4.28, 4.06, and 3.92 for 0, 5, 10, 15, 20, and 25 wt% recycled HDPE, respectively, with R² values between approximately 0.965 and 0.979. These fitted slopes are used here as experimental effective amplification measures rather than as intrinsic material properties.

**Fig 25:**
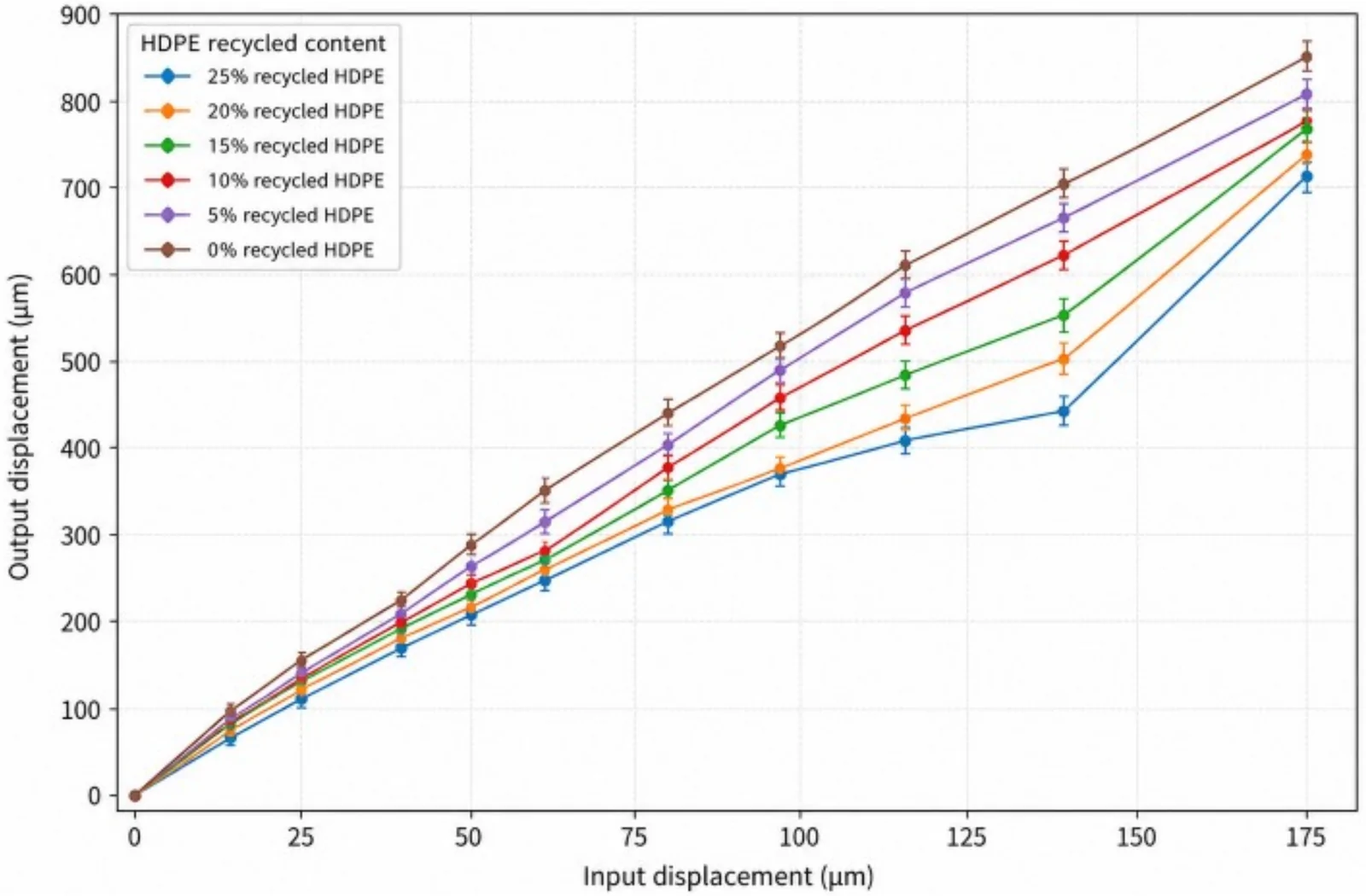
Experimental input–output displacement responses of bridge-type compliant mechanisms fabricated from HDPE with different recycled-HDPE fractions. Data are presented as mean ± SD (n = 3).

Despite the reduction in effective displacement amplification with increasing recycled-HDPE content, the three specimens tested for each composition exhibited similar output-displacement values, indicating acceptable short-term repeatability under the investigated fixture and loading protocol. Bridge-type compliant mechanisms are commonly analyzed using elastic-beam and energy-based formulations in which amplification depends on geometry as well as the translational and rotational stiffness of the flexure members [15–17,50,51]. The analytical studies of Ling et al. [50] and Zhu et al. [51] further show that bridge-type amplification can deviate from ideal kinematic predictions when flexure stiffness and geometric nonlinearity are considered. In the present study, elastic modulus and viscoelastic properties were not measured for each recycled-HDPE blend; therefore, a material-specific analytical prediction is not introduced. The measured slopes and endpoint ratios are instead reported as experimental effective amplification metrics, and claims regarding strength, fatigue durability, or intrinsic stiffness are avoided.

### 3.5. Practical comparison with state-of-the-art technologies

Based on the benchmark and parametric results, the capability of the fluid-layer architecture must be clearly positioned within the landscape of current state-of-the-art mold temperature control technologies. Table 5 presents a multi-criteria synthesis, comparing the proposed method against prevalent systems in the literature, such as straight cooling channels, 3D conformal cooling, rapid heat cycle molding (RHCM), and gas/induction heating.

**Table 5:** Practical comparison of the proposed fluid-layer architecture with state-of-the-art mold temperature control technologies.

Technology	Heating rate	Spatial uniformity	Manufacturing complexity & cost	Suitability for compliant mechanisms
Conventional straight channel [23–27]	Relatively slow	Limited by channel-to-cavity distance; local gradients may occur	Low; conventional machining	Limited for thin flexure regions when temperature non-uniformity is significant
3D conformal cooling [28–38]	Potentially fast	High when channels closely follow the cavity	High; often requires metal AM and post-processing	Potentially high; not directly benchmarked in this study
Variothermal/ RHCM [39–44]	Fast surface heating	Depends on heating method and geometry	Higher system complexity; dedicated heating/cooling hardware	Application-dependent; not directly benchmarked in this study
Gas/ induction heating [45–49]	Very fast surface heating	Can be localized depending on source and geometry	Requires specialized auxiliary equipment	Application-dependent; not directly benchmarked in this study
Proposed fluid-layer	Fast under the investigated conditions	Improved relative to the straight-channel baseline	Conventionally machinable modular geometry	Promising for the bridge-type mechanism evaluated here

Table 5 positions the fluid-layer architecture as an intermediate design concept between conventional straight channels and fully additively manufactured conformal cooling systems. Compared with the representative straight-channel thermal baseline and the corresponding numerical benchmark, the fluid-layer architecture produced a higher and more spatially uniform cavity-surface temperature under the investigated conditions. The more uniform cavity temperature is expected to reduce asymmetric thermal histories across the flexure arms; however, stiffness uniformity was not measured directly in this study. Relative to conformal cooling systems reported in the literature, the proposed approach offers a simpler conventionally machined geometry; however, this study did not directly benchmark its thermal performance against a conformal-cooling mold. Conformal cooling commonly relies on metal additive manufacturing and may require additional post-processing and maintenance considerations, depending on channel geometry and fabrication route. Conversely, the modular insert design of the fluid layer is fully machinable via standard CNC milling, is easily dismantled for cleaning, and can be integrated directly into existing industrial mold bases. RHCM and induction-based systems can provide rapid surface heating, but their operating principles, energy requirements, and temperature distributions differ from those of the fluid-layer approach. Because these systems were not tested directly in the present work, the comparison should be regarded as literature-based rather than as an experimental performance ranking. Overall, the fluid-layer architecture combines a short local conduction path with a relatively large fluid-solid heat-transfer area and can be fabricated using conventional CNC machining. Under the conditions evaluated here, it provided a higher cavity-surface temperature and a lower longitudinal temperature difference than the straight-channel baseline. Broader techno-economic comparisons with conformal cooling and dynamic heating systems require dedicated cost, energy, and cycle-time analyses.

### 3.6. Scope and limitations

The principal experimental contribution of this study is thermal characterization of the proposed fluid-layer system together with a representative thermographic straight-channel baseline and short-term displacement testing of molded HDPE mechanisms. The straight-channel thermal dataset contains a representative heating sequence and therefore does not provide repeatability statistics. In addition, no new straight-channel injection-molding experiment was performed in this revision; the incomplete-filling/complete-filling contrast remains a numerical prediction. Dimensional metrology of hinge thickness, shrinkage, left–right symmetry, and warpage was not available and is therefore not used to support claims of dimensional accuracy.

The 40 s heating interval refers to mold preheating before melt injection and should not be interpreted as an additional 40 s residence time of the polymer melt. Nevertheless, mold-wall temperature and cooling history can influence HDPE crystallization and morphology. Differential scanning calorimetry, polarized-light microscopy, gel permeation chromatography, dynamic mechanical analysis, tensile testing, and cyclic fatigue testing were not performed in the present study. Consequently, the observed recycled-HDPE displacement trend is not attributed to a specific change in crystallinity, molecular weight, morphology, viscoelasticity, or fatigue strength. These measurements are important priorities for future process–structure–property studies.

## 4. Conclusion

This study developed a conventionally machinable fluid-layer-assisted mold temperature-control architecture and evaluated it using experimentally validated thermo-fluid modeling, representative straight-channel thermography, simulation-based thermal and filling benchmarks, and short-term displacement measurements of molded HDPE compliant mechanisms. For the fluid-layer validation cases, the transient thermo-fluid model showed reasonable agreement with infrared measurements, with an overall MAE of 5.01 °C and RMSE of 6.44 °C across six validation points.

Under the numerical benchmark condition of an 80 °C inlet temperature, 59.5 g/s mass flow rate, and 40 s heating, the fluid-layer model predicted an approximately 4.0 °C higher average cavity-surface temperature and a 28.6% smaller maximum longitudinal temperature difference than the straight-channel model. Independent thermography of a representative steel straight-channel insert under the same inlet-temperature and flow-rate conditions confirmed the direction of this thermal advantage: at 40 s, the measured central surface temperature was approximately 59.9 °C for the straight-channel insert and 76.3 °C for the fluid-layer insert, and the fluid-layer longitudinal profile was substantially flatter. Because the experimental straight-channel geometry was used as a representative conventional baseline, the experimental values are not interpreted as a one-to-one quantitative validation of the numerical +4.0 °C and 28.6% magnitudes.

Melt-filling simulations predicted incomplete filling of the thin flexure region for the straight-channel configuration and complete filling for the fluid-layer configuration under the evaluated numerical conditions; this filling comparison was not experimentally repeated for the straight-channel mold. For HDPE containing 0–25 wt% recycled material, the measured input–output displacement response remained approximately linear over the investigated range, while the effective amplification decreased as recycled content increased. At an input displacement of approximately 175 µm, the mean output displacement decreased from 857 to 745 µm, corresponding to a 13.1% reduction. These measurements demonstrate repeatable short-term displacement response but do not establish fatigue durability, tensile strength, viscoelastic behavior, or polymer-structure changes. Future work should therefore combine repeated production-cycle thermal measurements, experimental straight-channel molding, dimensional metrology, polymer morphology characterization, and cyclic mechanical testing.
