# Supplementary material for: Fluid-layer-assisted mold temperature control for injection-molded bridge-type compliant mechanisms: Experimental thermal validation and simulation-based benchmarking against conventional cooling channels
Source: PLoS One. 2026 Oct 27;21(10):e0361383. doi: 10.1371/journal.pone.0361383 (PMC13712607; doi:10.1371/journal.pone.0361383)
Supplement: Supplementary file 2 [file pone.0361383.s002.docx]

**Response to Reviewer #3**

*Fluid-Layer-Assisted Mold Temperature Control for Injection-Molded Bridge-Type Compliant Mechanisms: Experimental Thermal Validation and Simulation-Based Benchmarking Against Conventional Cooling Channels*

We thank the reviewer for the careful and constructive assessment. The manuscript has been substantially revised. The principal change is that the thermal advantage of the fluid-layer architecture is now supported by an additional representative experimental straight-channel thermography baseline, while the melt-filling comparison is explicitly retained as simulation-based. Our point-by-point responses are provided below.

**Comment 1.** The injection and packing pressures in Table 2 appear too low for melt pressure.

**Response:** We agree that the original terminology was ambiguous. The values 26 bar and 23 bar were recorded from the Haitian machine controller as filling- and packing-pressure settings; they are not direct measurements of nozzle or cavity melt pressure. Table 2 and the Methods now explicitly call them “machine-controller filling-pressure setting” and “machine-controller packing-pressure setting.” The approximately 40.6 MPa value reported later in the paper is a predicted melt pressure from the filling simulation and is clearly distinguished from the controller settings.

**Changes in the revised manuscript:** Revised Section 2.5.5, Table 2, and Section 3.4.

**Comment 2.** Where is the gate in Figure 8?

**Response:** The Methods now explicitly identify the central sprue/gate position on the mechanism midline and state that this is the symmetric feed position used in the molding/filling analysis. The Figure 8 description and surrounding text were revised so the feed location is no longer implicit.

**Changes in the revised manuscript:** Revised Section 2.5.4 and Figure 8 discussion.

**Comment 3.** Provide a diagram for the displacement measurement and identify the input and output blocks.

**Response:** A compact measurement schematic has been added to Section 2.5.6. It identifies the two symmetric input indicators and input blocks, the central output block and output indicator, and the screw-driven loading fixture. The text also clarifies zeroing, alignment, incremental loading, averaging of the two input readings, and the use of three specimens per composition.

**Changes in the revised manuscript:** Revised Section 2.5.6.

**Comment 4.** What injection-molding machine was used?

**Response:** The archived controller photographs identify the machine manufacturer as Haitian Plastics Machinery. The revised manuscript now reports this manufacturer. The specific model designation is not legible in the retained records, so we have not inserted an unverified model number. The controller photographs also support the reported processing settings in Table 2.

**Changes in the revised manuscript:** Revised Section 2.5.4.

**Comment 5.** Were the temperature measurements made during the injection-molding process? If only mold temperature was measured, the experiment may be too simple.

**Response:** We have clarified the experimental sequence. Thermography used for thermal-model validation and architecture benchmarking was intentionally conducted during the controlled pre-injection mold-heating stage, without polymer in the cavity. This isolates the thermal response of the mold/fluid system from the transient heat input of the injected melt and enables direct comparison with the thermo-fluid model. The injection-molding trials are a separate application stage. To strengthen the thermal evidence, the revision additionally includes an actual conventional straight-channel steel-insert thermography baseline under the same 80 °C inlet and 59.5 g/s flow condition. We do not claim that the thermography represents the entire injection cycle.

**Changes in the revised manuscript:** Revised Sections 2.5.2–2.5.3, Section 3.2, Figs. 12–13, and Section 3.6.

Thank you again for your careful review and valuable comments. Your suggestions have helped us improve the clarity, methodology, and presentation of the manuscript.

Yours sincerely,

Pham Son Minh - Corresponding author Ho Chi Minh City University of Technology and Engineering

E-mai: minhps@hcmute.edu.vn
