# Supplementary material for: Fluid-layer-assisted mold temperature control for injection-molded bridge-type compliant mechanisms: Experimental thermal validation and simulation-based benchmarking against conventional cooling channels
Source: PLoS One. 2026 Oct 27;21(10):e0361383. doi: 10.1371/journal.pone.0361383 (PMC13712607; doi:10.1371/journal.pone.0361383)
Supplement: Supplementary file 3 [file pone.0361383.s001.docx]

**10 August 2026**

**RESPONSE TO PREVIOUS EDITORIAL COMMENTS**

*PLOS ONE resubmission history: PONE-D-26-08292 → PONE-D-26-39514 → present resubmission*

**Manuscript:** Fluid-Layer-Assisted Mold Temperature Control for Injection-Molded Bridge-Type Compliant Mechanisms: Benchmarking Against Conventional Cooling Channels

We thank the PLOS ONE editorial team for the previous guidance. The manuscript has been substantially revised to address the scientific concerns raised for PONE-D-26-08292. We also clarify below the subsequent administrative history of PONE-D-26-39514.

**Submission history and disclosure**

**PONE-D-26-08292:** The manuscript was declined because the research question was considered insufficiently defined and the benchmarking of the proposed mold-temperature-control architecture against current approaches was considered inadequate.

**PONE-D-26-39514:** After substantial scientific revision, the manuscript was resubmitted. However, the earlier PONE-D-26-08292 interaction was inadvertently omitted from the cover letter. The submission was therefore not considered further. No new scientific concerns were raised in that decision. We apologize for this administrative oversight. The present submission explicitly declares both prior interactions.

**Editorial Concern 1 — Clearly defined research question and contribution**

**Concern:** The previous decision indicated that the manuscript did not sufficiently satisfy PLOS ONE’s expectation for original research addressing a clearly defined research question and contributing to the academic knowledge base.

**Response:** We agree and have substantially reframed the manuscript around a specific comparative research objective. The revised Introduction now asks, in substantive terms, whether the fluid-layer-assisted mold architecture provides measurable thermal and molding advantages over a conventional straight-channel design under equivalent operating conditions. The revised study evaluates cavity-surface temperature during transient heating, spatial temperature uniformity, thin-flexure filling capability, and the displacement response of the molded bridge-type compliant mechanism. Table 1 now explicitly identifies the state-of-the-art gap and positions the proposed architecture relative to existing mold temperature-control approaches.

**Changes in the manuscript:** Title; Abstract; Introduction and Table 1; Results and Discussion; Conclusion.

**Editorial Concern 2 — Benchmarking against current approaches**

**Concern:** The previous decision stated that a newly designed tool or method should be placed in context through adequate benchmarking against current state-of-the-art approaches, and that the comparisons in the previous version were insufficient.

**Response:** We agree and have added a direct benchmark against a conventional straight-channel configuration under identical boundary conditions: inlet temperature 80 °C, mass flow rate 59.5 g/s, and heating time 40 s. The benchmark is simulation-based and uses the thermal model that was experimentally validated by infrared thermography. Under these conditions, the fluid-layer architecture increases the average cavity-surface temperature by approximately 4.0 °C and reduces the maximum longitudinal temperature difference, ΔTmax, by 28.6% relative to the straight-channel baseline. The melt-filling analysis further predicts incomplete filling at the thin flexure hinge for the straight-channel configuration and complete filling for the fluid-layer configuration.

The revised manuscript also adds a separate practical comparison with conformal cooling, RHCM/variothermal systems, and gas/induction heating. We have deliberately distinguished these literature-based comparisons from the direct straight-channel benchmark and do not present them as experimental performance rankings. The manuscript also states that energy consumption, pumping requirements, and broader techno-economic comparisons were not quantified in this study.

**Changes in the manuscript:** Section 3.2; Tables 6 and 7; Figure 11; Section 3.4 and Figure 18 for the filling comparison; Section 3.5 and Table 8; Conclusion.

**Administrative correction for PONE-D-26-39514**

The present cover letter now explicitly declares both PONE-D-26-08292 and PONE-D-26-39514. We have included this response document and a revision-marked manuscript so that the editorial team can readily identify how the original scientific concerns were addressed. We again apologize for the previous omission of the submission history.

We respectfully hope that these revisions and the complete disclosure of the submission history address the concerns raised and allow the manuscript to proceed to peer review.

Sincerely yours,

**Pham Son Minh**

Corresponding Author

Faculty of Mechanical Engineering

HCM City University of Technology and Engineering, Ho Chi Minh City, Vietnam

Email: minhps@hcmute.edu.vn
