# Supplementary material for: Fluid-layer-assisted mold temperature control for injection-molded bridge-type compliant mechanisms: Experimental thermal validation and simulation-based benchmarking against conventional cooling channels
Source: PLoS One. 2026 Oct 27;21(10):e0361383. doi: 10.1371/journal.pone.0361383 (PMC13712607; doi:10.1371/journal.pone.0361383)
Supplement: Peer review history

# Author comment: Author response to Decision Letter 0

**DOI:** 10.1371/journal.pone.0361383.r001

10 Aug 2026

Attachment Submitted filename: 4_Response_to_Previous_Editorial_Comments_PLOS_ONE.docx

# Peer review: Decision Letter 0

**DOI:** 10.1371/journal.pone.0361383.r002

**Authors:** Khalil Khalil

31 Aug 2026

Dear Dr. Son Minh,

Thank you for submitting your manuscript to PLOS One. After careful consideration, we feel that it has merit but does not fully meet PLOS One’s publication criteria as it currently stands. Therefore, we invite you to submit a revised version of the manuscript that addresses the points raised during the review process.

Please submit your revised manuscript by Oct 30 2026 11:59PM. If you will need more time than this to complete your revisions, please reply to this message or contact the journal office at plosone@plos.org. When you're ready to submit your revision, log on to https://www.editorialmanager.com/pone/ and select the 'Submissions Needing Revision' folder to locate your manuscript file.

- A letter that responds to each point raised by the academic editor and reviewer(s). You should upload this letter as a separate file labeled 'Response to Reviewers'.
- A marked-up copy of your manuscript that highlights changes made to the original version. You should upload this as a separate file labeled 'Revised Manuscript with Track Changes'.
- An unmarked version of your revised paper without tracked changes. You should upload this as a separate file labeled 'Manuscript'.

If you would like to make changes to your financial disclosure, please include your updated statement in your cover letter. Guidelines for resubmitting your figure files are available below the reviewer comments at the end of this letter.

If applicable, we recommend that you deposit your laboratory protocols in protocols.io to enhance the reproducibility of your results. Protocols.io assigns your protocol its own identifier (DOI) so that it can be cited independently in the future. For instructions see: https://journals.plos.org/plosone/s/submission-guidelines#loc-laboratory-protocols. Additionally, PLOS One offers an option for publishing peer-reviewed Lab Protocol articles, which describe protocols hosted on protocols.io. Read more information on sharing protocols at https://plos.org/protocols?utm_medium=editorial-email&utm_source=authorletters&utm_campaign=protocols.

As the corresponding author, your ORCID iD is verified in the submission system and will appear in the published article. PLOS supports the use of ORCID, and we encourage all coauthors to register for an ORCID iD and use it as well. Please encourage your coauthors to verify their ORCID iD within the submission system before final acceptance, as unverified ORCID iDs will not appear in the published article. *Only*  the individual author can complete the verification step; PLOS staff *cannot*  verify ORCID iDs on behalf of authors.

We look forward to receiving your revised manuscript.

Kind regards,

Khalil Abdelrazek Khalil, Ph.D.

Academic Editor

PLOS One

Journal Requirements:

When submitting your revision, we need you to address these additional requirements.

1. Please ensure that your manuscript meets PLOS ONE's style requirements, including those for file naming. The PLOS ONE style templates can be found at

https://journals.plos.org/plosone/s/file?id=wjVg/PLOSOne_formatting_sample_main_body.pdf and

https://journals.plos.org/plosone/s/file?id=ba62/PLOSOne_formatting_sample_title_authors_affiliations.pdf

2. Please note that PLOS One has specific guidelines on code sharing for submissions in which author-generated code underpins the findings in the manuscript. In these cases, we expect all author-generated code to be made available without restrictions upon publication of the work. Please review our guidelines at https://journals.plos.org/plosone/s/materials-and-software-sharing#loc-sharing-code and ensure that your code is shared in a way that follows best practice and facilitates reproducibility and reuse.

3. Thank you for stating the following in the Acknowledgments Section of your manuscript:

“This research was funded by Ho Chi Minh City University of Technology and Engineering, Vietnam, project grant No.: T2025-35.”

We note that you have provided funding information that is not currently declared in your Funding Statement. However, funding information should not appear in the Acknowledgments section or other areas of your manuscript. We will only publish funding information present in the Funding Statement section of the online submission form.

Please remove any funding-related text from the manuscript and let us know how you would like to update your Funding Statement. Currently, your Funding Statement reads as follows:

“This research was funded by Ho Chi Minh City University of Technology and Engineering, Vietnam, project grant No.: T2025-35”

Please include your amended statements within your cover letter; we will change the online submission form on your behalf.

4. We note that the grant information you provided in the ‘Funding Information’ and ‘Financial Disclosure’ sections do not match.

When you resubmit, please ensure that you provide the correct grant numbers for the awards you received for your study in the ‘Funding Information’ section.

5. Thank you for stating the following financial disclosure:

“This research was funded by Ho Chi Minh City University of Technology and Engineering, Vietnam, project grant No.: T2025-35”

Please state what role the funders took in the study.  If the funders had no role, please state: "The funders had no role in study design, data collection and analysis, decision to publish, or preparation of the manuscript."

If this statement is not correct you must amend it as needed.

Please include this amended Role of Funder statement in your cover letter; we will change the online submission form on your behalf.

6. Thank you for stating the following financial disclosure:

“This research was funded by Ho Chi Minh City University of Technology and Engineering, Vietnam, project grant No.: T2025-35”

We note that one or more of the authors is affiliated with the funding organization, indicating the funder may have had some role in the design, data collection, analysis or preparation of your manuscript for publication; in other words, the funder played an indirect role through the participation of the co-authors. If the funding organization did not play a role in the study design, data collection and analysis, decision to publish, or preparation of the manuscript and only provided financial support in the form of authors' salaries and/or research materials, please do the following:

1. Review your statements relating to the author contributions, and ensure you have specifically and accurately indicated the role(s) that these authors had in your study. These amendments should be made in the online form.

2. Confirm in your cover letter that you agree with the following statement, and we will change the online submission form on your behalf:

“The funder provided support in the form of salaries for authors [insert relevant initials], but did not have any additional role in the study design, data collection and analysis, decision to publish, or preparation of the manuscript. The specific roles of these authors are articulated in the ‘author contributions’ section.

7. Please upload a new copy of Figures 10, 11, 18, 19 and 20 as the detail is not clear. Please follow the link for more information:  https://journals.plos.org/plosone/s/figures

8. We note that Figures 7, 9, 18, 19 and Table 3 and 4 in your submission contain copyrighted images. All PLOS content is published under the Creative Commons Attribution License (CC BY 4.0), which means that the manuscript, images, and Supporting Information files will be freely available online, and any third party is permitted to access, download, copy, distribute, and use these materials in any way, even commercially, with proper attribution. For more information, see our copyright guidelines: http://journals.plos.org/plosone/s/licenses-and-copyright.

We require you to either (1) present written permission from the copyright holder to publish these figures specifically under the CC BY 4.0 license, or (2) remove the figures from your submission:

1. You may seek permission from the original copyright holder of Figure(s) [#] to publish the content specifically under the CC BY 4.0 license.

We recommend that you contact the original copyright holder with the Content Permission Form (http://journals.plos.org/plosone/s/file?id=7c09/content-permission-form.pdf) and the following text:

“I request permission for the open-access journal PLOS ONE to publish XXX under the Creative Commons Attribution License (CCAL) CC BY 4.0 (http://creativecommons.org/licenses/by/4.0/). Please be aware that this license allows unrestricted use and distribution, even commercially, by third parties. Please reply and provide explicit written permission to publish XXX under a CC BY license and complete the attached form.”

Please upload the completed Content Permission Form or other proof of granted permissions as an "Other" file with your submission.

In the figure caption of the copyrighted figure, please include the following text: “Reprinted from [ref] under a CC BY license, with permission from [name of publisher], original copyright [original copyright year].”

2. If you are unable to obtain permission from the original copyright holder to publish these figures under the CC BY 4.0 license or if the copyright holder’s requirements are incompatible with the CC BY 4.0 license, please either i) remove the figure or ii) supply a replacement figure that complies with the CC BY 4.0 license. Please check copyright information on all replacement figures and update the figure caption with source information. If applicable, please specify in the figure caption text when a figure is similar but not identical to the original image and is therefore for illustrative purposes only.

9. We note you have included a table to which you do not refer in the text of your manuscript. Please ensure that you refer to Table 2 in your text; if accepted, production will need this reference to link the reader to the Table.

10. If the reviewer comments include a recommendation to cite specific previously published works, please review and evaluate these publications to determine whether they are relevant and should be cited. There is no requirement to cite these works unless the editor has indicated otherwise.

[Note: HTML markup is below. Please do not edit.]

Reviewers' comments:

Reviewer's Responses to Questions

**Comments to the Author**

1. Is the manuscript technically sound, and do the data support the conclusions?

Reviewer #1: Yes

Reviewer #2: Partly

Reviewer #3: Yes

**********

2. Has the statistical analysis been performed appropriately and rigorously?

Reviewer #1: Yes

Reviewer #2: N/A

Reviewer #3: Yes

**********

3. Have the authors made all data underlying the findings in their manuscript fully available?

The PLOS Data policy

Reviewer #1: Yes

Reviewer #2: Yes

Reviewer #3: No

**********

4. Is the manuscript presented in an intelligible fashion and written in standard English?

Reviewer #1: Yes

Reviewer #2: No

Reviewer #3: Yes

**********

Reviewer #1: This study evaluated the effectiveness of Fluid-Layer-Assisted Mold Temperature Control (FL-AMTC) in the injection molding of bridge-type compliant mechanisms by comparing it with conventional direct cooling channels. With this thermal distribution homogenization, full filling of the flexure hinge section—where short shots (under-filling) previously occurred—was predicted. In light of polymer science and processing technology knowledge, this approach is theoretical and analytical regarding: (1) design guidelines for improving transcription and thermal control methods in precision molding of polymers, and (2) heat transfer homogenization and hinge filling in simulations. However, there is a lack of: (1) experimental validation via actual machines, (2) consideration of the effects of heating cycles and thermal history on the primary/secondary polymer structures (degree of crystallinity, morphology, molecular weight degradation, etc.), and (3) evaluation regarding mechanical strength (fatigue strength and viscoelastic behavior of the flexure hinge) of the molded product. Validation and discussion based on this polymer science approach are essential for manuscript acceptance.

Quantitative validation data using an actual mold and injection molding machine should be presented for the results obtained in the simulation, such as "4.0 °C increase in cavity surface temperature" and "28.6 % reduction in ΔTmax," showing complete filling at the flexure hinge. Numerical analysis alone may not fully reflect the actual thermal response of the mold or the wall slip of the polymer melt.

Please clarify how the temperature change on the cavity surface due to 40 seconds of heating by the fluid layer affects the morphology of the polymer material (resin)—such as degree of crystallinity and spherulite size for crystalline resins, or orientation and residual stress for amorphous resins—using DSC (Differential Scanning Calorimetry) and polarized light microscopy. Furthermore, please address the possibility of thermal decomposition or molecular weight reduction (via GPC measurement, etc.) of the polymer associated with an extended thermal history.

The subject of this study is the bridge-type compliant mechanism (hinge section). Please verify whether the molded hinge section possesses not only the resolution of short shots, but also the intended flexibility, mechanical strength, and cyclic fatigue durability through viscoelasticity measurements (DMA), tensile testing, or fatigue testing.

Regarding Table 1 added in the revision, please add items for "Cycle Time," "Mold Durability," and "Manufacturing Cost" in addition to the comparison with Conformal Cooling and RHCM/Variothermal, to clarify the academic and industrial positioning.

Please thoroughly ensure in the Abstract and Conclusion that the results are clearly stated as simulation results (e.g., "Numerical simulations revealed that..."), and revise expressions to avoid confusion with experimental results.

Reviewer #2: His work develops a conventionally machinable fluid-layer-assisted mold temperature-control architecture for injection molding of bridge-type compliant mechanisms. The study combines transient conjugate thermo-fluid simulation, infrared-thermography validation, a simulation-based benchmark against straight cooling channels, melt-filling analysis, and displacement tests of virgin/recycled-HDPE mechanisms. Under the reported benchmark condition, the fluid-layer design raises the average cavity-surface temperature by about 4.0 °C, reduces the maximum longitudinal temperature difference by 28.6%, and predicts complete filling of the thin flexure hinges. The molded mechanisms also retain an approximately linear input-output displacement response for recycled-HDPE fractions up to 25 wt%. However, several aspects of the modeling, experimental validation, and interpretation of the compliant-mechanism response require further clarification and strengthening before the conclusions can be fully supported.

1. Add an experimental straight-channel control for the core benchmark in Section 3.2, at least for representative surface-temperature profiles and molded-part filling. At present, the reported +4.0 °C, 28.6% reduction in ΔTmax, and short-shot/complete-filling contrast are derived from simulations for the baseline architecture, so the comparative claim is stronger than the experimental evidence.

2. State the complete thermo-fluid formulation in Section 2.4, including the governing equations, flow regime or turbulence treatment, inlet/outlet conditions, external convection coefficient, temperature-dependent material properties, and the treatment of insert-plate contact resistance. The description of “fully developed flow” and the use of mass flow rate to determine convective heat transfer need enough detail for independent reproduction.

3. Provide a quantitative mesh- and time-step-independence study rather than only stating that grid convergence was verified. Report at least three discretization levels and the convergence of average cavity temperature and ΔTmax; also recheck the node/element counts reported in Fig. 5 for consistency.

4. Separate the influence of fluid-layer thickness from the accompanying change in mean velocity and convective conditions in Section 3.3.

5. Introduce a mechanics-based prediction of the bridge mechanism and compare it with Fig. 20, rather than relying only on empirical input-output curves. Report the displacement amplification ratio and relate the measured trend to geometry and material stiffness. Established analytical treatments such as “Enhanced mathematical modeling of the displacement amplification ratio for piezoelectric compliant mechanisms” (M. Ling et al., Smart Materials and Structures, 25(7), 075022) and “Linear and nonlinear analytical equations for fast design of three types of triangular-amplified compliant mechanisms” (J. Zhu et al., Precision Engineering, 86, 342–350) provide suitable reference points for the formulation and validation strategy. A brief discussion of these representative formulations would also help clarify the theoretical basis and positioning of the present analysis.

6. Add dimensional metrology of the molded compliant mechanisms, particularly flexure-hinge thickness, left-right symmetry, shrinkage/warpage, and short-shot occurrence. Also report the displacement-indicator resolution and accuracy, fixture compliance, alignment procedure, and loading protocol; otherwise, narrow the claims regarding dimensional/mechanical consistency to what the present displacement data directly demonstrate.

Reviewer #3: This manuscript studies fluid-layer-assisted mold temperature control for injection-molded, bridge-type compliant mechanisms, benchmarking it against conventional cooling channels. This is an interesting paper related to injection mold cooling systems; however, I have several comments as follows:

1. Injection and packing pressures are too low in Table 2, The injection pressure must be at least 1000 bar (10 MPa) to drive the melt into cavity.

2. Where is the gate on Figure 8?

3. Can you provide a diagram for Section 2.5.5 Displacement measurement of the compliant mechanisms? Where is the input and output blocks?

4. What injection molding machine was used?

5. Do you run an injection molding process during all temperature measurements? These measurements must be conducted during the process to verify the heating and cooling functions. If it is just a mold temperature measurement, this is too simple of an experiment.

**********

what does this mean?). If published, this will include your full peer review and any attached files.

If you choose “no”, your identity will remain anonymous but your review may still be made public.

**Do you want your identity to be public for this peer review?**  For information about this choice, including consent withdrawal, please see our Privacy Policy

Reviewer #1: No

Reviewer #2: No

Reviewer #3: **Yes:**  shyh-shin hwang

**********

[NOTE: If reviewer comments were submitted as an attachment file, they will be attached to this email and accessible via the submission site. Please log into your account, locate the manuscript record, and check for the action link "View Attachments". If this link does not appear, there are no attachment files.]

To ensure your figures meet our technical requirements, please review our figure guidelines: https://journals.plos.org/plosone/s/figures

You may also use PLOS’s free figure tool, NAAS, to help you prepare publication quality figures: https://journals.plos.org/plosone/s/figures#loc-tools-for-figure-preparation.

NAAS will assess whether your figures meet our technical requirements by comparing each figure against our figure specifications.

# Author comment: Author response to Decision Letter 1

**DOI:** 10.1371/journal.pone.0361383.r003

30 Sep 2026

Response to Reviewer #1

Fluid-Layer-Assisted Mold Temperature Control for Injection-Molded Bridge-Type Compliant Mechanisms: Experimental Thermal Validation and Simulation-Based Benchmarking Against Conventional Cooling Channels

We thank the reviewer for the careful and constructive assessment. The manuscript has been substantially revised. The principal change is that the thermal advantage of the fluid-layer architecture is now supported by an additional representative experimental straight-channel thermography baseline, while the melt-filling comparison is explicitly retained as simulation-based. Our point-by-point responses are provided below.

Comment 1. Experimental validation using an actual mold/machine is insufficient; the +4.0 °C, 28.6% reduction in ΔTmax, and filling claims should be supported experimentally.

Response: We agree that the original wording did not sufficiently distinguish experimentally validated thermal behavior from simulation-based benchmarking. We therefore added an experimental thermal baseline using a conventional straight-channel steel insert. The straight-channel insert was tested with the same inlet-water temperature (80 °C) and mass flow rate (59.5 g/s) used for the benchmark condition. Radiometric Fluke TiS20 IS2 data were reprocessed at the registered cavity center and along the same 70-mm longitudinal molding span used for the fluid-layer analysis. At 40 s, the representative experimental central temperature was approximately 59.9 °C for the straight-channel insert and 76.3 °C for the fluid-layer insert. The longitudinal line mean was approximately 57.9 °C and 75.6 °C, respectively, and the corresponding line temperature ranges were approximately 12.4 °C and 1.5 °C. These data experimentally confirm the direction of the thermal advantage. We also explicitly state that the numerical +4.0 °C and 28.6% values remain simulation results and that the experimental straight-channel geometry is a representative conventional baseline rather than an exact replica of the numerical geometry. No new straight-channel injection-molding trial was performed; consequently, the short-shot/complete-filling comparison remains a simulation prediction and is now labeled as such throughout.

Changes in the revised manuscript: Abstract; new Section 2.5.3; revised Section 3.2; revised Table 7; new Figs. 12 and 13; Section 3.6; Conclusion.

Comment 2. Please investigate the effect of mold heating/thermal history on polymer morphology, crystallinity, spherulite size, molecular orientation/residual stress, and molecular-weight degradation using DSC, polarized microscopy, GPC, or equivalent methods.

Response: We agree that these measurements would be valuable for a process–structure–property study. However, they were not part of the archived experimental campaign and were not generated for this revision. We have therefore narrowed the scope rather than attributing the measured displacement behavior to unmeasured molecular mechanisms. The revised manuscript clarifies that the reported 40 s heating interval is mold preheating before injection, not an additional 40 s melt-residence time. We also state explicitly that DSC, polarized-light microscopy, GPC, and related morphology/molecular-weight measurements were not performed and that the recycled-HDPE displacement trend cannot be assigned to crystallinity, morphology, or molecular-weight degradation from the present data.

Changes in the revised manuscript: New Section 3.6 and revised Abstract/Conclusion; speculative polymer-structure statements were removed or qualified.

Comment 3. Please verify flexibility, mechanical strength, viscoelastic behavior, and cyclic fatigue durability of the molded flexure hinges using DMA, tensile, or fatigue testing.

Response: We agree that the displacement experiment alone cannot establish strength, viscoelasticity, or fatigue durability. We have therefore changed the terminology from broad “mechanical performance/stability” claims to “short-term input–output displacement response” and “effective displacement amplification.” Figure 22 now reports mean ± SD (n = 3), and the revised text quantifies the endpoint amplification ratio and the slopes of linear fits. The endpoint ratio decreased from approximately 4.90 for virgin HDPE to 4.26 at 25 wt% recycled HDPE. The fitted effective slopes decreased from approximately 4.88 to 3.92, with R² values of approximately 0.965–0.979. These are presented as experimental response metrics, not as strength or fatigue properties. DMA, tensile, and cyclic fatigue testing are explicitly identified as future work.

Changes in the revised manuscript: Revised Section 3.4, Fig. 22 caption, new Section 3.6, and Conclusion.

Comment 4. Add Cycle Time, Mold Durability, and Manufacturing Cost to the technology comparison.

Response: Table 1 has been restructured to include cycle-time implication, mold durability/maintenance, and manufacturing cost for straight channels, conformal cooling, RHCM/variothermal, gas/induction heating, and the proposed fluid-layer architecture. Where the present study did not quantify a metric, the table states “not quantified” instead of assigning unsupported numerical values.

Changes in the revised manuscript: Revised Table 1.

Comment 5. Ensure the Abstract and Conclusion clearly identify simulation results and do not confuse them with experimental results.

Response: We agree and have revised the manuscript consistently. The +4.0 °C average-temperature increase and 28.6% reduction in the numerical ΔTmax metric are explicitly identified as simulation results. The newly added straight-channel thermography is described separately as representative experimental evidence confirming the direction of the thermal advantage. The short-shot/complete-filling comparison is explicitly described as melt-filling simulation, not experimental validation.

Changes in the revised manuscript: Revised title, Abstract, Sections 3.2 and 3.4, Table 7, Section 3.6, and Conclusion.

Thank you again for your careful review and valuable comments. Your suggestions have helped us improve the clarity, methodology, and presentation of the manuscript.

Yours sincerely,

Pham Son Minh - Corresponding author Ho Chi Minh City University of Technology and Engineering

E-mai: minhps@hcmute.edu.vn

----------------------------------------------------------------

Response to Reviewer #2

Fluid-Layer-Assisted Mold Temperature Control for Injection-Molded Bridge-Type Compliant Mechanisms: Experimental Thermal Validation and Simulation-Based Benchmarking Against Conventional Cooling Channels

We thank the reviewer for the careful and constructive assessment. The manuscript has been substantially revised. The principal change is that the thermal advantage of the fluid-layer architecture is now supported by an additional representative experimental straight-channel thermography baseline, while the melt-filling comparison is explicitly retained as simulation-based. Our point-by-point responses are provided below.

Comment 1. Add an experimental straight-channel control for representative surface-temperature profiles and molded-part filling.

Response: We have added a representative experimental straight-channel thermal control using the steel insert under the same 80 °C inlet-water temperature and 59.5 g/s mass-flow condition used for the benchmark. The revised paper now includes direct center-point and 40-s longitudinal-profile comparisons between the straight-channel and fluid-layer steel inserts. This substantially strengthens the experimental support for the thermal comparison. We did not perform a new straight-channel molding campaign; therefore the filling contrast is still explicitly presented as simulation-based, and the manuscript no longer implies that straight-channel short shots were experimentally observed.

Changes in the revised manuscript: New Section 2.5.3; revised Section 3.2; Table 7; new Figs. 12–13; Section 3.6; Conclusion.

Comment 2. State the complete thermo-fluid formulation, including governing equations, flow treatment, boundary conditions, material properties, and contact resistance; clarify the role of mass flow rate and convective heat transfer.

Response: We expanded the numerical-method description with the governing conservation equations for mass, momentum, fluid energy, and solid heat conduction, together with temperature/heat-flux continuity at the fluid–solid interface. The revised text clarifies that mass flow rate is imposed as an inlet-flow condition and controls convective transport through the resolved fluid domain; it is not treated as an independently imposed interface heat-transfer coefficient. The inlet temperature, outlet condition, external convective heat loss, and thermally continuous insert/cooling-plate interface are also stated. A separate experimentally measured contact-resistance value was not available in the archived model, so no such value is invented. The archived source files also do not preserve a complete solver-specific turbulence-closure record; this limitation is stated explicitly rather than assigning an undocumented model.

Changes in the revised manuscript: New Section 2.4.1 and revised Section 2.4.

Comment 3. Provide quantitative mesh- and time-step-independence results using at least three discretization levels and recheck node/element counts.

Response: No new simulations were performed for this revision, and the archived dataset available to us does not contain a documented three-level mesh/time-step independence study. We therefore removed the previous unsupported statement that formal grid independence had been quantitatively verified. The revised manuscript states the local mesh-refinement strategy but explicitly identifies quantitative discretization sensitivity as a limitation requiring additional numerical work. This change avoids presenting unverified convergence evidence.

Changes in the revised manuscript: Revised Section 2.4 and new Section 2.4.1.

Comment 4. Separate the effect of fluid-layer thickness from the accompanying changes in flow area, mean velocity, and convective conditions.

Response: We agree. Section 3.3 has been rewritten to distinguish insert thickness from fluid-layer thickness. The revised discussion states that insert thickness mainly controls solid conduction resistance, whereas changing fluid-layer thickness at fixed mass flow simultaneously changes cross-sectional area, mean velocity, hydraulic conditions, and convective transport. The observed thickness trend is therefore no longer attributed solely to a shorter conduction distance.

Changes in the revised manuscript: Revised Section 3.3.

Comment 5. Add a mechanics-based prediction/interpretation of the bridge mechanism and report the displacement amplification ratio, with reference to the analytical literature suggested by the reviewer.

Response: We added a quantitative mechanics-oriented interpretation without introducing an unsupported material-specific prediction. The endpoint amplification ratio δout/δin is now reported, decreasing from approximately 4.90 to 4.26 as recycled-HDPE content increases from 0 to 25 wt%. Linear fits to the measured mean curves give effective slopes of approximately 4.88, 4.74, 4.55, 4.28, 4.06, and 3.92, with R² values of approximately 0.965–0.979. We added the analytical bridge-type references by Ling et al. and Zhu et al. and discuss the roles of flexure geometry, translational/rotational stiffness, and geometric nonlinearity. Because elastic modulus and viscoelastic properties were not measured for each recycled-HDPE blend, we do not present a material-specific analytical prediction that would require unmeasured inputs.

Changes in the revised manuscript: Revised Section 3.4 and new Refs. 50–51.

Comment 6. Add dimensional metrology and provide indicator resolution/accuracy, fixture compliance, alignment, and loading details; otherwise narrow the claims.

Response: The archived dataset does not contain systematic hinge-thickness, shrinkage, warpage, left–right symmetry, fixture-compliance, or instrument-accuracy calibration data. We therefore narrowed the claims to what the displacement data directly support. The measurement protocol is now described more explicitly: two input indicators are positioned symmetrically, input displacement is the mean of the two input readings, the indicators are zeroed after mounting/alignment, displacement is applied incrementally by the screw-driven fixture, and three specimens are tested for each HDPE composition. A compact measurement schematic has also been added in Section 2.5.6. Dimensional accuracy and intrinsic stiffness are not claimed.

Changes in the revised manuscript: Revised Section 2.5.6, Section 3.4, and Section 3.6.

Thank you again for your careful review and valuable comments. Your suggestions have helped us improve the clarity, methodology, and presentation of the manuscript.

Yours sincerely,

Pham Son Minh - Corresponding author Ho Chi Minh City University of Technology and Engineering

E-mai: minhps@hcmute.edu.vn

------------------------------------------------------------------

Response to Reviewer #3

Fluid-Layer-Assisted Mold Temperature Control for Injection-Molded Bridge-Type Compliant Mechanisms: Experimental Thermal Validation and Simulation-Based Benchmarking Against Conventional Cooling Channels

We thank the reviewer for the careful and constructive assessment. The manuscript has been substantially revised. The principal change is that the thermal advantage of the fluid-layer architecture is now supported by an additional representative experimental straight-channel thermography baseline, while the melt-filling comparison is explicitly retained as simulation-based. Our point-by-point responses are provided below.

Comment 1. The injection and packing pressures in Table 2 appear too low for melt pressure.

Response: We agree that the original terminology was ambiguous. The values 26 bar and 23 bar were recorded from the Haitian machine controller as filling- and packing-pressure settings; they are not direct measurements of nozzle or cavity melt pressure. Table 2 and the Methods now explicitly call them “machine-controller filling-pressure setting” and “machine-controller packing-pressure setting.” The approximately 40.6 MPa value reported later in the paper is a predicted melt pressure from the filling simulation and is clearly distinguished from the controller settings.

Changes in the revised manuscript: Revised Section 2.5.5, Table 2, and Section 3.4.

Comment 2. Where is the gate in Figure 8?

Response: The Methods now explicitly identify the central sprue/gate position on the mechanism midline and state that this is the symmetric feed position used in the molding/filling analysis. The Figure 8 description and surrounding text were revised so the feed location is no longer implicit.

Changes in the revised manuscript: Revised Section 2.5.4 and Figure 8 discussion.

Comment 3. Provide a diagram for the displacement measurement and identify the input and output blocks.

Response: A compact measurement schematic has been added to Section 2.5.6. It identifies the two symmetric input indicators and input blocks, the central output block and output indicator, and the screw-driven loading fixture. The text also clarifies zeroing, alignment, incremental loading, averaging of the two input readings, and the use of three specimens per composition.

Changes in the revised manuscript: Revised Section 2.5.6.

Comment 4. What injection-molding machine was used?

Response: The archived controller photographs identify the machine manufacturer as Haitian Plastics Machinery. The revised manuscript now reports this manufacturer. The specific model designation is not legible in the retained records, so we have not inserted an unverified model number. The controller photographs also support the reported processing settings in Table 2.

Changes in the revised manuscript: Revised Section 2.5.4.

Comment 5. Were the temperature measurements made during the injection-molding process? If only mold temperature was measured, the experiment may be too simple.

Response: We have clarified the experimental sequence. Thermography used for thermal-model validation and architecture benchmarking was intentionally conducted during the controlled pre-injection mold-heating stage, without polymer in the cavity. This isolates the thermal response of the mold/fluid system from the transient heat input of the injected melt and enables direct comparison with the thermo-fluid model. The injection-molding trials are a separate application stage. To strengthen the thermal evidence, the revision additionally includes an actual conventional straight-channel steel-insert thermography baseline under the same 80 °C inlet and 59.5 g/s flow condition. We do not claim that the thermography represents the entire injection cycle.

Changes in the revised manuscript: Revised Sections 2.5.2–2.5.3, Section 3.2, Figs. 12–13, and Section 3.6.

Thank you again for your careful review and valuable comments. Your suggestions have helped us improve the clarity, methodology, and presentation of the manuscript.

Yours sincerely,

Pham Son Minh - Corresponding author Ho Chi Minh City University of Technology and Engineering

E-mai: minhps@hcmute.edu.vn

Attachment Submitted filename: Response to Reviewers 3.docx

# Peer review: Decision Letter 1

**DOI:** 10.1371/journal.pone.0361383.r004

**Authors:** Khalil Khalil

12 Oct 2026

FLUID-LAYER-ASSISTED MOLD TEMPERATURE CONTROL FOR INJECTION-MOLDED BRIDGE-TYPE COMPLIANT MECHANISMS: EXPERIMENTAL THERMAL VALIDATION AND SIMULATION-BASED BENCHMARKING AGAINST CONVENTIONAL COOLING CHANNELS

PONE-D-26-40146R1

Dear Dr. Son Minh,

We’re pleased to inform you that your manuscript has been judged scientifically suitable for publication and will be formally accepted for publication once it meets all outstanding technical requirements.

Within one week, you’ll receive an e-mail detailing the required amendments. When these have been addressed, you’ll receive a formal acceptance letter and your manuscript will be scheduled for publication.

An invoice will be generated when your article is formally accepted. Please note, if your institution has a publishing partnership with PLOS and your article meets the relevant criteria, all or part of your publication costs will be covered. Please make sure your user information is up-to-date by logging into Editorial Manager at Editorial Manager® and clicking the ‘Update My Information' link at the top of the page. For questions related to billing, please contact billing support.

If your institution or institutions have a press office, please notify them about your upcoming paper to help maximize its impact. If they’ll be preparing press materials, please inform our press team as soon as possible -- no later than 48 hours after receiving the formal acceptance. Your manuscript will remain under strict press embargo until 2 pm Eastern Time on the date of publication. For more information, please contact onepress@plos.org.

Kind regards,

Khalil Abdelrazek Khalil, Ph.D.

Academic Editor

PLOS One

Additional Editor Comments (optional):

Reviewers' comments:

Reviewer's Responses to Questions

**Comments to the Author**

Reviewer #1: All comments have been addressed

Reviewer #2: All comments have been addressed

Reviewer #3: All comments have been addressed

**********

2. Is the manuscript technically sound, and do the data support the conclusions?

Reviewer #1: Yes

Reviewer #2: Yes

Reviewer #3: Yes

**********

3. Has the statistical analysis been performed appropriately and rigorously?

Reviewer #1: Yes

Reviewer #2: N/A

Reviewer #3: Yes

**********

4. Have the authors made all data underlying the findings in their manuscript fully available?

The PLOS Data policy

Reviewer #1: Yes

Reviewer #2: Yes

Reviewer #3: Yes

**********

5. Is the manuscript presented in an intelligible fashion and written in standard English?

Reviewer #1: Yes

Reviewer #2: Yes

Reviewer #3: Yes

**********

Reviewer #1: This paper has accurately addressed the points raised by the reviewers. Therefore, as it stands, it is worthy of publication in this journal.

Reviewer #2: The authors have revised the manuscript. The manuscript could be accepted in its present form. As a choice, some tables are recommended to be revised as figures.

Reviewer #3: Thank you for your revisions. Authors must pay attention to the maximum injection pressure of the injection molding. This is a very important process parameter for injection molding.

**********

what does this mean?). If published, this will include your full peer review and any attached files.

If you choose “no”, your identity will remain anonymous but your review may still be made public.

**Do you want your identity to be public for this peer review?**  For information about this choice, including consent withdrawal, please see our Privacy Policy

Reviewer #1: No

Reviewer #2: No

Reviewer #3: **Yes:**  Shyh-shin Hwang

**********

# Editor report: Acceptance letter

**DOI:** 10.1371/journal.pone.0361383.r005

**Authors:** Khalil Khalil

PONE-D-26-40146R1

PLOS One

Dear Dr. Son Minh,

I'm pleased to inform you that your manuscript has been deemed suitable for publication in PLOS One. Congratulations! Your manuscript is now being handed over to our production team.

At this stage, our production department will prepare your paper for publication. This includes ensuring the following:

* All references, tables, and figures are properly cited

* All relevant supporting information is included in the manuscript submission,

* There are no issues that prevent the paper from being properly typeset

You will receive further instructions from the production team, including instructions on how to review your proof when it is ready. Please keep in mind that we are working through a large volume of accepted articles, so please give us a few days to review your paper and let you know the next and final steps.

Lastly, if your institution or institutions have a press office, please let them know about your upcoming paper now to help maximize its impact. If they'll be preparing press materials, please inform our press team within the next 48 hours. Your manuscript will remain under strict press embargo until 2 pm Eastern Time on the date of publication. For more information, please contact onepress@plos.org.

You will receive an invoice from PLOS for your publication fee after your manuscript has reached the completed accept phase. If you receive an email requesting payment before acceptance or for any other service, this may be a phishing scheme. Learn how to identify phishing emails and protect your accounts at https://explore.plos.org/phishing.

If we can help with anything else, please email us at customercare@plos.org.

Thank you for submitting your work to PLOS One and supporting open access.

Kind regards,

PLOS One Editorial Office Staff

on behalf of

Dr. Khalil Abdelrazek Khalil

Academic Editor

PLOS One
